# Zoonotic Tick-Borne Pathogens in Temperate and Cold Regions of Europe—A Review on the Prevalence in Domestic Animals

**DOI:** 10.3389/fvets.2020.604910

**Published:** 2020-12-10

**Authors:** Andrea Springer, Antje Glass, Anna-Katharina Topp, Christina Strube

**Affiliations:** Institute for Parasitology, Centre for Infection Medicine, University of Veterinary Medicine Hannover, Hanover, Germany

**Keywords:** *Borrelia*, *Rickettsia*, *Anaplasma*, *Babesia*, *Neoehrlichia mikurensis*, tick-borne encephalitis, tick-borne diseases, vector-borne diseases

## Abstract

Ticks transmit a variety of pathogens affecting both human and animal health. In temperate and cold regions of Europe (Western, Central, Eastern, and Northern Europe), the most relevant zoonotic tick-borne pathogens are tick-borne encephalitis virus (TBEV), *Borrelia* spp. and *Anaplasma phagocytophilum*. More rarely, *Rickettsia* spp., *Neoehrlichia mikurensis*, and zoonotic *Babesia* spp. are identified as a cause of human disease. Domestic animals may also be clinically affected by these pathogens, and, furthermore, can be regarded as sentinel hosts for their occurrence in a certain area, or even play a role as reservoirs or amplifying hosts. For example, viraemic ruminants may transmit TBEV to humans via raw milk products. This review summarizes the role of domestic animals, including ruminants, horses, dogs, and cats, in the ecology of TBEV, *Borrelia* spp., *A. phagocytophilum, Rickettsia* spp., *N. mikurensis*, and zoonotic *Babesia* species. It gives an overview on the (sero-)prevalence of these infectious agents in domestic animals in temperate/cold regions of Europe, based on 148 individual prevalence studies. Meta-analyses of seroprevalence in asymptomatic animals estimated an overall seroprevalence of 2.7% for TBEV, 12.9% for *Borrelia burgdorferi* sensu lato (s.l.), 16.2% for *A. phagocytophilum* and 7.4% for *Babesia divergens*, with a high level of heterogeneity. Subgroup analyses with regard to animal species, diagnostic test, geographical region and decade of sampling were mostly non-significant, with the exception of significantly lower *B. burgdorferi* s.l. seroprevalences in dogs than in horses and cattle. More surveillance studies employing highly sensitive and specific test methods and including hitherto non-investigated regions are needed to determine if and how global changes in terms of climate, land use, agricultural practices and human behavior impact the frequency of zoonotic tick-borne pathogens in domestic animals.

## Introduction

Many tick-borne diseases (TBDs) are so-called meta-zoonoses, i.e., they may be transmitted to humans as well as animals via their invertebrate tick host (1). In temperate/cold regions of Europe, the hard tick *Ixodes ricinus* is the most important vector of TBDs in terms of both animal and human (public) health, followed by *Dermacentor reticulatus* and *Dermacentor marginatus* (2). Meta-zoonotic pathogens transmitted by *I. ricinus* include tick-borne encephalitis virus (TBEV), *Borrelia* spp., *Anaplasma phagocytophilum, Rickettsia* spp., *Neoehrlichia mikurensis* and zoonotic *Babesia* spp., while ticks of the genus *Dermacentor* may transmit *Rickettsia* spp. to both animals and humans, among others (3). In addition, *D. reticulatus* is now also recognized as a vector for TBEV (4).

*Ixodes ricinus* has a broad host spectrum, including birds, various wild and domestic animals as well as humans, and occurs in a wide variety of habitats throughout Europe, as far north as 66° N (Norway), close to the Arctic Circle (5). The species' range has continuously expanded northward as well as into higher altitudes during the past decades, probably driven by climatic and environmental changes (6, 7). Similarly, the distribution of *D. reticulatus* is expanding in several European countries (8, 9). A northward spread has been documented in the Baltic countries (10) as well as in Germany (11), but the species has not been documented in Scandinavia to date. In contrast, the distribution of *D. marginatus* seems to be comparatively stable with a northern distribution limit at ~51° N in central Germany (11). In addition to the range expansion of different tick species, changes in human behavior toward more outdoor activities increase the risk of tick bites (2).

In consequence, the incidence and public health burden of TBDs seem to be increasing in Europe. Lyme borreliosis (LB), caused by spirochaetes of the *Borrelia burgdorferi* sensu lato (s.l.) complex and transmitted primarily by *I. ricinus*, is the most common TBD in humans in the Northern Hemisphere. In Europe, annual incidence rates vary between 0.001 and 464 cases/100,000 inhabitants (12). In the Netherlands, a more than 2-fold increase of medical consultations and hospital admissions due to LB was noted from 1994 to 2005 (13). A similar increase in diagnosed LB cases was observed in the United Kingdom from 1998 to 2016 (14). Likewise, the incidence of tick-borne encephalitis, a flavivirus infection transmitted also primarily by *I. ricinus*, has increased significantly since the year 1990 in several European countries (15). The geographic pattern of this disease is also changing, with new transmission foci emerging in previously unaffected regions and countries, e.g., in the Netherlands in 2016 (16) and in the United Kingdom in 2018 (17).

In addition to rising disease incidences, several new tick-borne pathogens have been described in recent decades (18). Although known already since 1995 (19), pathogenicity of *Borrelia miyamotoi*, a tick-transmitted relapsing-fever spirochaete, was first reported in 2011 (20). Similarly, *N. mikurensis* was isolated from ticks and mammals (21) years before being recognized as a human (22) and probably veterinary (23) pathogen. Furthermore, the list of emerging zoonotic tick-borne pathogens relevant in Europe includes several *Rickettsia* spp. (18) and *Babesia* spp. (24). *Borrelia miyamotoi, N. mikurensis, Rickettsia helvetica* and the relevant zoonotic *Babesia* spp. are all transmitted by *I. ricinus*, which constitutes the main vector of zoonotic TBDs in central and northern Europe. In contrast, *D. marginatus*, the vector of *Rickettsia slovaca*, has a rather limited geographic distribution, and *D. reticulatus*, the vector of *Rickettsia raoultii*, rarely bites humans (11). Therefore, these tick species are of minor importance regarding zoonotic infections.

Domestic animals may also be clinically affected by these pathogens, and, furthermore, can be regarded as sentinel hosts for their occurrence in a certain area, or even play a role as reservoir hosts. Additionally, viraemic ruminants may directly transmit TBEV to humans via raw milk products, causing large outbreaks (25). In this review, we summarize the role of domestic animals, including ruminants, horses, dogs, and cats, in the ecology of TBEV, *Borrelia* spp., *A. phagocytophilum, Rickettsia* spp., *N. mikurensis*, and zoonotic *Babesia* species. Unlike for humans, no systematic surveillance of TBDs in domestic animals exists, making it difficult to assess whether the patterns of increasing disease incidence observed in humans can also be found in other species. Therefore (sero-)prevalence data on the mentioned pathogens in domestic animals in temperate and cold regions of Europe are compiled to analyze temporal and regional trends, the influence of the utilized diagnostic test and to identify knowledge gaps requiring further attention.

## Methods

### Literature Survey

**Figure 1:**
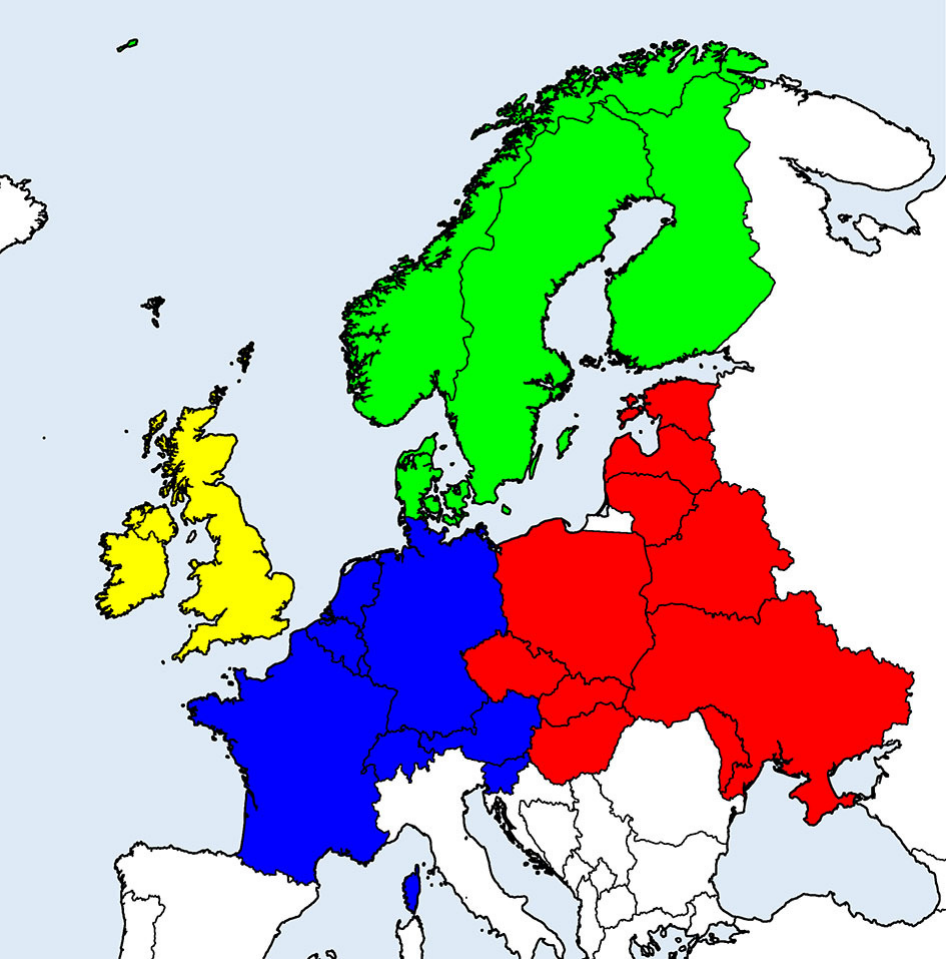
European countries considered in the literature survey on (sero-)prevalence of tick-borne zoonotic diseases in domestic animals. Geographical regions are indicated by different colors: Scandinavia (green), British Isles (yellow), Western continental (blue), and Eastern continental Europe (red).

Systematic literature search on (sero-)prevalence data in temperate and cold regions of Europe [Northern, Western, Central, and Eastern Europe (excluding Russia); see Figure 1 for included countries] was conducted in the PubMed database in May and July 2020, using combinations of the term “prevalence” with each of “animals,” “ruminants,” “horses,” “dogs,” “cats” and each of “TBEV,” “*Borrelia*,” “*Anaplasma*,” “*Rickettsia*,” “*Neoehrlichia*,” and “*Babesia*.”

Further records were obtained by searching the bibliographies of relevant articles and via incidental findings using other databases, e.g., Google Scholar. Original publications in English and different national languages (e.g., German, French, if available) were included. Articles that did not refer to the considered geographical region, did not contain (sero-)prevalence data, e.g., clinical case reports, or that presented data from only one herd/flock, were excluded.

### Meta-Analyses

For TBEV, *B. burgdorferi* s.l., *A. phagocytophilum* and *Babesia divergens* seroprevalence, data based on healthy/asymptomatic animals or randomly selected diagnostic samples were subjected to meta-analyses, to gain a comprehensive picture on TBD prevalence in the general domestic animal population. As the number of studies retrieved for the remaining pathogens was low, no meta-analyses were conducted. If studies reported data on healthy and symptomatic groups, only data referring to the healthy group were extracted, because seroprevalences in symptomatic animals may be higher than in the general population.

Random-effects meta-analysis of proportions was conducted with the package “meta” (v. 4.13-0) (26) in R v. 4.0.2 (27), using the inverse variance method with logit transformation and restricted maximum likelihood estimation of the between-study variance (τ^2^). To assess heterogeneity between studies, *Q*-tests were performed and the *I*^2^ statistic was assessed, with values ≥50% considered heterogeneous. To evaluate possible sources of heterogeneity, subgroup analyses were performed according to animal species, type of diagnostic test used, geographical region and decade of sampling. For analyses according to geographical region, the considered countries were classified into Eastern or Western continental Europe, Scandinavia or British Isles (Figure 1). In cases when studies did not report the period of sampling, it was assumed that the decade of sampling corresponded to the decade of data publication. In subgroup analyses, a common τ^2^ was assumed across subgroups.

If studies reported more than one seroprevalence rate, referring to different species, geographic regions, or data acquisition periods, these were considered separately. Since observational prevalence studies are unlikely to suffer from publication bias, i.e., low prevalence rates have a similar probability of being published as higher prevalence rates (28), no assessment of publication bias was performed.

## Results

**Figure 2:**
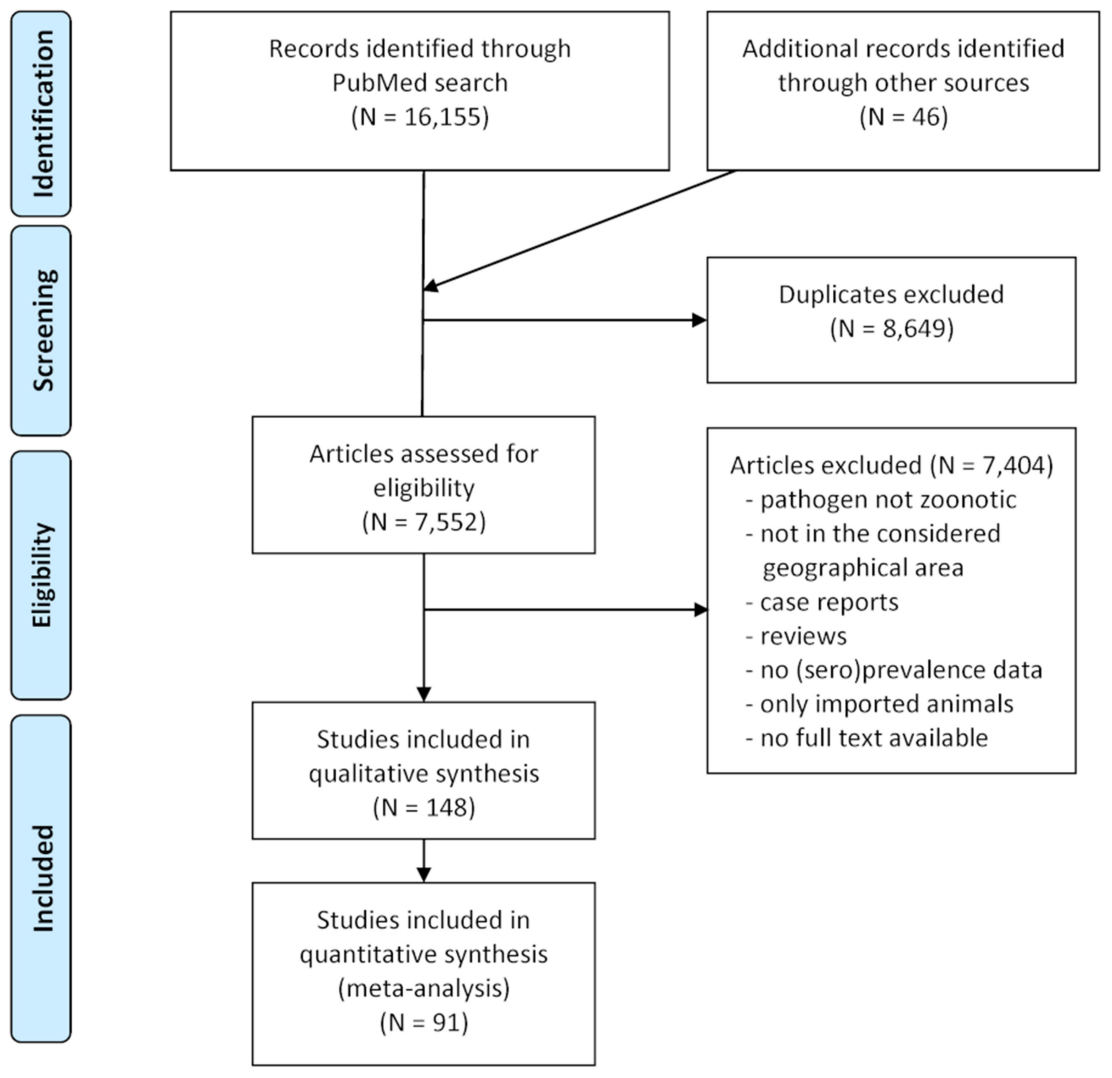
PRISMA flow-chart detailing the selection process applied during the literature survey.

In total, 7,552 publications were assessed for eligibility and 7,404 were excluded because they were not relevant with regard to the considered pathogens or geographical range, consisted of clinical case reports referring to single animals or herds, dealt only with imported animals or did not contain sufficient data. The selection process during the literature survey is depicted in Figure 2, with a final dataset containing 148 articles. Of these, 65 reported data on *A. phagocytophilum*, 55 on *B. burgdorferi* s.l., 35 on TBEV, 18 on zoonotic *Babesia* species, 9 on *Rickettsia* spp., and 5 on *N. mikurensis*. Some publications contained data on more than one of these pathogens.

**Figure 3:**
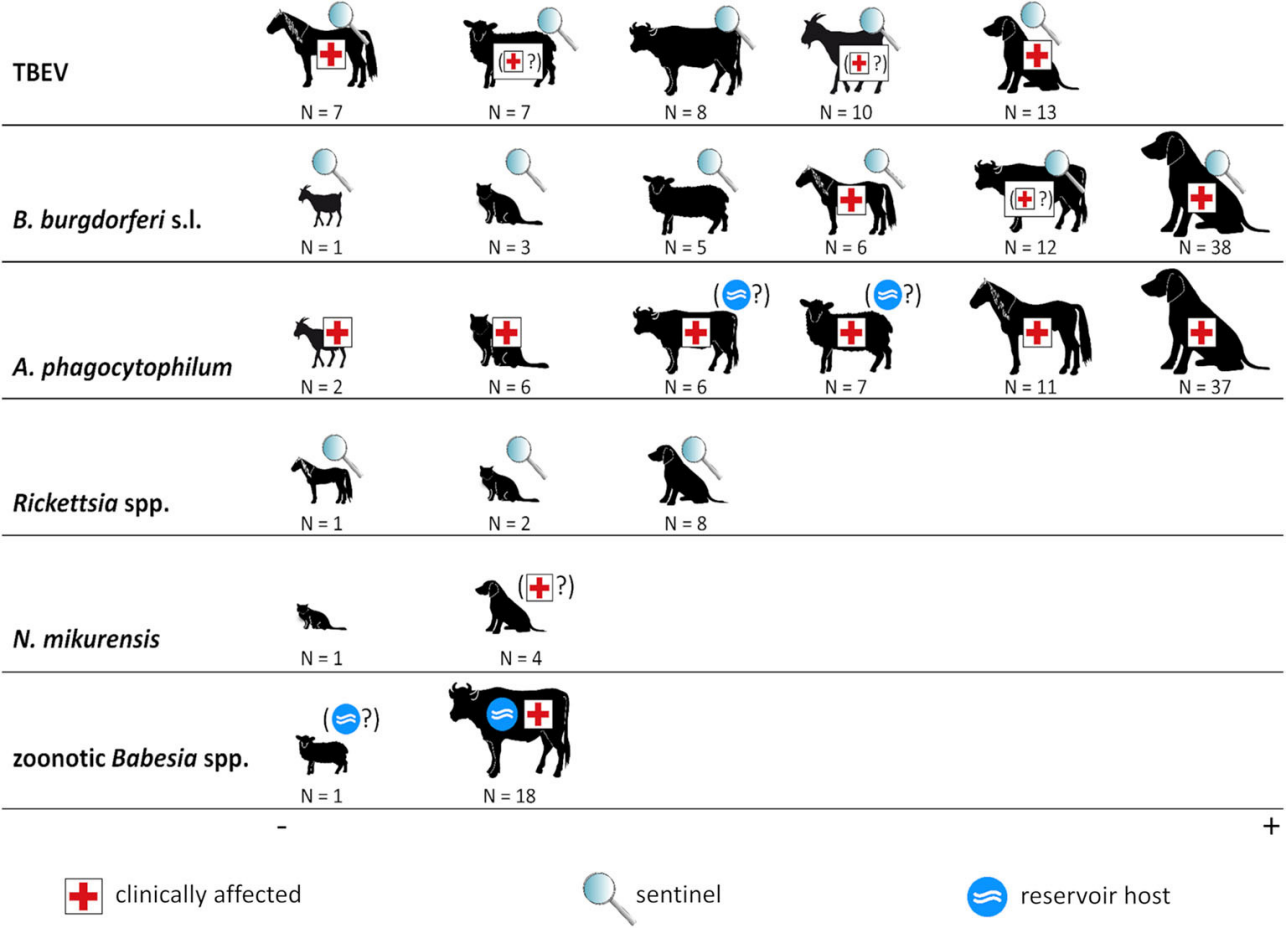
Animal species and tick-borne pathogens. The size of animal icons corresponds to the number of studies. Symbols indicate the role of each animal species in the ecology of the respective pathogen. Symbols in brackets indicate that this role is uncertain. *Borrelia miyamotoi* is not shown as no (sero-)prevalence studies regarding this pathogen in domestic animals were retrieved.

An overview of the roles of domestic animals regarding the considered tick-borne pathogens is given in Figure 3. In the following, these roles as well as the (sero-)prevalence rates are discussed in detail for each infectious agent.

### Tick-Borne Encephalitis Virus

Tick-borne encephalitis is regarded as the most important arthropod-borne viral disease in Europe (29). It is caused by a flavivirus which is mainly transmitted by *I. ricinus*, but the vector potential of *D. reticulatus* has also been shown (4). Unlike other tick-associated pathogens, it is not consistently distributed throughout the range of its vectors, but occurs in a patchy pattern in delimited geographic areas, termed microfoci or “hotspots.” In these foci, it circulates between rodents and ticks and occasionally spills over to domestic animals and humans (29). In recent decades, a geographical spread of the virus has been observed in Europe with new transmission foci having recently emerged in the Netherlands (16) and the United Kingdom (17).

TBEV may cause severe neurologic disease in humans, horses, dogs, and probably also in ruminants (Figure 3). Furthermore, most domestic animals are regarded as useful sentinels for human TBE risk (30), with the exception of cats for which no data exist, explaining the comparatively large number of retrieved studies.

**Figure 4:**
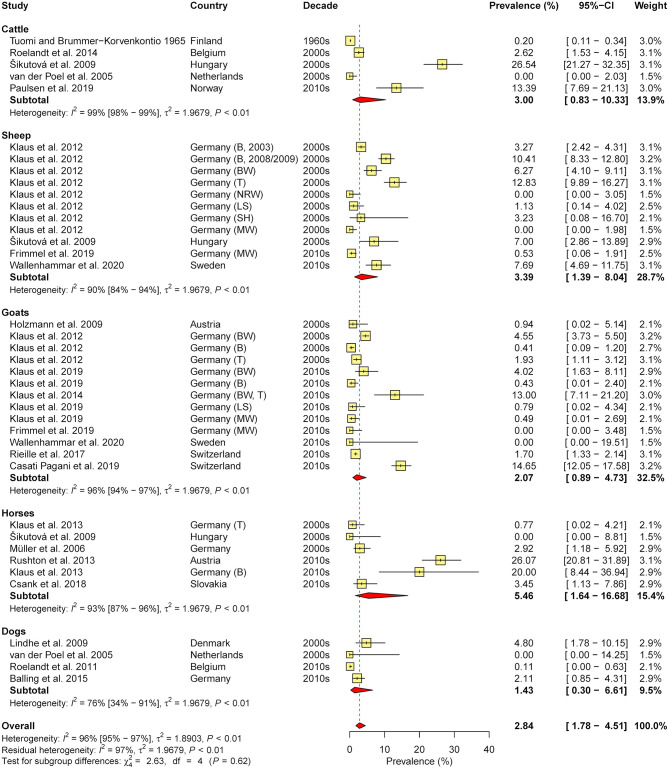
Forest plot displaying the results of random-effects meta-analysis of tick-borne encephalitis virus seroprevalence in domestic animals, with subgroup analysis according to animal species. Individual study results are shown as yellow squares, corresponding in size to the weight of the study on the overall prevalence estimate. Error bars indicate 95% confidence intervals (CI). Pooled prevalences are shown as red diamonds and the red dotted vertical line indicates the estimated overall prevalence.

In total, 36 studies were retrieved (eight containing data on cattle, seven on sheep, 10 on goats, seven on horses, and 13 on dogs). As cross-reactions with other flaviviruses (e.g., West Nile virus, Louping Ill virus) in serological tests are common, only studies which confirmed positive samples via seroneutralisation test (SNT), considered the gold standard of TBEV serology (31), were included in the meta-analysis of seroprevalence (*N* = 20, with 39 animal cohorts). Therefore, subgroup analysis according to diagnostic test was not performed. The estimated overall prevalence was 2.8%, with a significant level of heterogeneity (*I*^2^ = 95.8%; 95% CI: 95.0–96.5%; *P* < 0.001). No significant differences between animal species (χ^2^ = 2.6, df = 4, *P* = 0.622; Figure 4) nor between decades (χ^2^ = 4.4, df = 2, *P* = 0.110) or regions (χ^2^ = 1.9, df = 2, *P* = 0.390) were found.

#### Ruminants

Domestic ruminants develop viraemia upon TBEV infection, which usually lasts a few days, but remain mostly asymptomatic (32). However, they excrete TBEV in milk during the viraemic phase, potentially leading to human infection via raw milk products like unpasteurized milk or raw milk cheese. In goat milk, infective virus can be detected for up to 19 days post infection (33). Such alimentary transmission often causes clusters of cases [e.g., (34, 35)] and is regarded as the second most important route of human infection (25).

In addition, a few clinical cases of neurologic disease in small ruminants due to TBEV have been described (36, 37). Possibly, clinical cases in these species are often overlooked or misinterpreted, e.g., as *Listeria monocytogenes* infection (36), and may be more common than previously thought. Furthermore, ruminants are regarded as useful sentinel species for TBEV occurrence. They have a comparatively restricted range of activity, i.e., they travel less than dogs and horses, and show persistence of antibodies for up to 28 months post infection (38). Therefore, several studies have been conducted on TBEV seroprevalence in domestic ruminants, particularly in goats and sheep (Supplementary Table 1).

Studies on cattle (*N* = 8, two from Norway, one each from Belgium, Finland, Hungary, Lithuania, the Netherlands, and Poland) reported seroprevalences ranging from 0.0% in the Netherlands (39) to 26.5% in Hungary (40) (Supplementary Table 1). One Norwegian study assessed TBEV excretion in milk and found 5.4% PCR-positive samples (41).

Seven studies reported seroprevalence data on sheep (two each from Sweden and Germany, one each from Hungary, Lithuania, and Slovakia), with values ranging from 0.0% in northern Germany (42) to 25.6% in farms with high lamb morbidity and mortality in Sweden (43). However, no confirmation of positive samples by SNT was performed in the latter study, so that cross-reactions with other flavivirus infections, e.g., louping ill, which has previously been detected in Norwegian sheep (44), cannot be ruled out. Seroprevalence in the 10 studies reporting data on goats (four from Germany, one each from Austria, Lithuania, the Netherlands, and Poland) ranged from 0.0% in the northern German federal state of Mecklenburg-Western Pomerania, which is not regarded as a TBEV risk area (45), to 14.6% in the Swiss canton of Ticino (46). Direct pathogen detection in sheep and goats, e.g., by PCR, was not reported in the considered studies.

#### Horses

As in ruminants, TBEV infections in horses are mostly asymptomatic (47). However, cases of encephalomyelitis with symptoms such as anorexia, ataxia, spasms, and epileptic seizures have been described (48, 49). Mild neurologic deficits may persist after recovery (49).

The literature survey resulted in the identification of seven studies on TBEV seroprevalence in horses (three from Germany, two from Austria, one each from Hungary and Slovakia; Supplementary Table 1), with values ranging from 0.0% in Hungary (40) to 33.0% in a TBEV risk area in Bavaria, Germany (49). All of these studies confirmed positive samples by SNT (Supplementary Table 1).

#### Dogs and Cats

In dogs, a similar TBE disease course as in humans with severe, often fatal neurological manifestations due to encephalitis has been described (50). However, high seroprevalence rates in some areas indicate that only a small proportion of infected dogs develops disease, whereas most infections remain asymptomatic (47). Because dogs usually accompany their owners, they are regarded as valuable sentinels for human TBEV risk. However, as companions of man, they often have a travel history, which makes assessment of TBE risk in a certain area based on dog sera less reliable compared to other sentinel animals (30).

In the present survey, 13 studies presenting data on TBEV (sero-)prevalence in dogs were identified (three from Germany, two from the Czech Republic, one each from Austria, Belgium, Denmark, Finland, the Netherlands, Norway, and Poland, and one study reporting data on dogs from different European countries; Supplementary Table 1). Seroprevalence ranged from 0.0% in the Netherlands (39) to 53.6% in dogs with neurological signs in Germany (51). However, positive results were not confirmed by SNT in the latter study, so that the possibility of cross-reactions with other flaviviruses needs to be considered. Cross-reactions also appear probable in light of the high seroprevalence detected in healthy dogs (30.4%) in the same study, compared with an estimated overall seroprevalence of 1.4% based on asymptomatic dogs when positive samples were confirmed by SNT (Figure 4). Nevertheless, another study including dogs with neurological illness determined a TBEV infection rate of 12.6% by real-time PCR (52), indicating that TBEV prevalence among dogs with neurologic disease may be substantial.

No cases of TBEV infections in cats have been published to date. Preliminary data of a study including more than 200 cats from a TBE-endemic area in Germany showed no seropositive individuals (personal communication with Martin Pfeffer, Institute of Animal Hygiene and Veterinary Public Health, University of Leipzig, and Gerhard Dobler, Bundeswehr Institute of Microbiology, Munich).

### *Borrelia burgdorferi* s.l.

The *B. burgdorferi* s.l. complex currently comprises 22 recognized species of spirochaetal bacteria (53), at least nine of which occur in European tick populations (54). Of those, *Borrelia afzelii* and *Borrelia garinii* are the most prevalent and constitute the most important agents of human LB throughout Europe (54, 55). *Borrelia burgdorferi* s.l. predominantly circulates between *Ixodes* ticks (in Europe *I. ricinus*) and wild mammals (e.g., *B. afzelii, B. bavariensis*), birds (e.g., *B. garinii, B. valaisiana*), or reptiles (*B. lusitaniae*) as reservoir hosts (56).

In total, 53 studies reporting *B. burgdorferi* s.l. (sero-)prevalence rates in domestic animals were retrieved (Supplementary Table 2). Since a vaccine against borreliosis in dogs was introduced to the European market at the end of the 1990s, it is important to distinguish between vaccinated and naturally exposed dogs when evaluating seroprevalence (57). Antibodies against the variable major protein-like sequence expressed (VlsE) and one of its invariable regions, the C6 peptide, as well as the outer surface protein OspF indicate natural exposure, because these antigens are not present in the available vaccines (58). Therefore, dog seroprevalence studies conducted after 1995 were only considered in the meta-analysis if they were based on a C6 or OspF assay.

**Figure 5:**
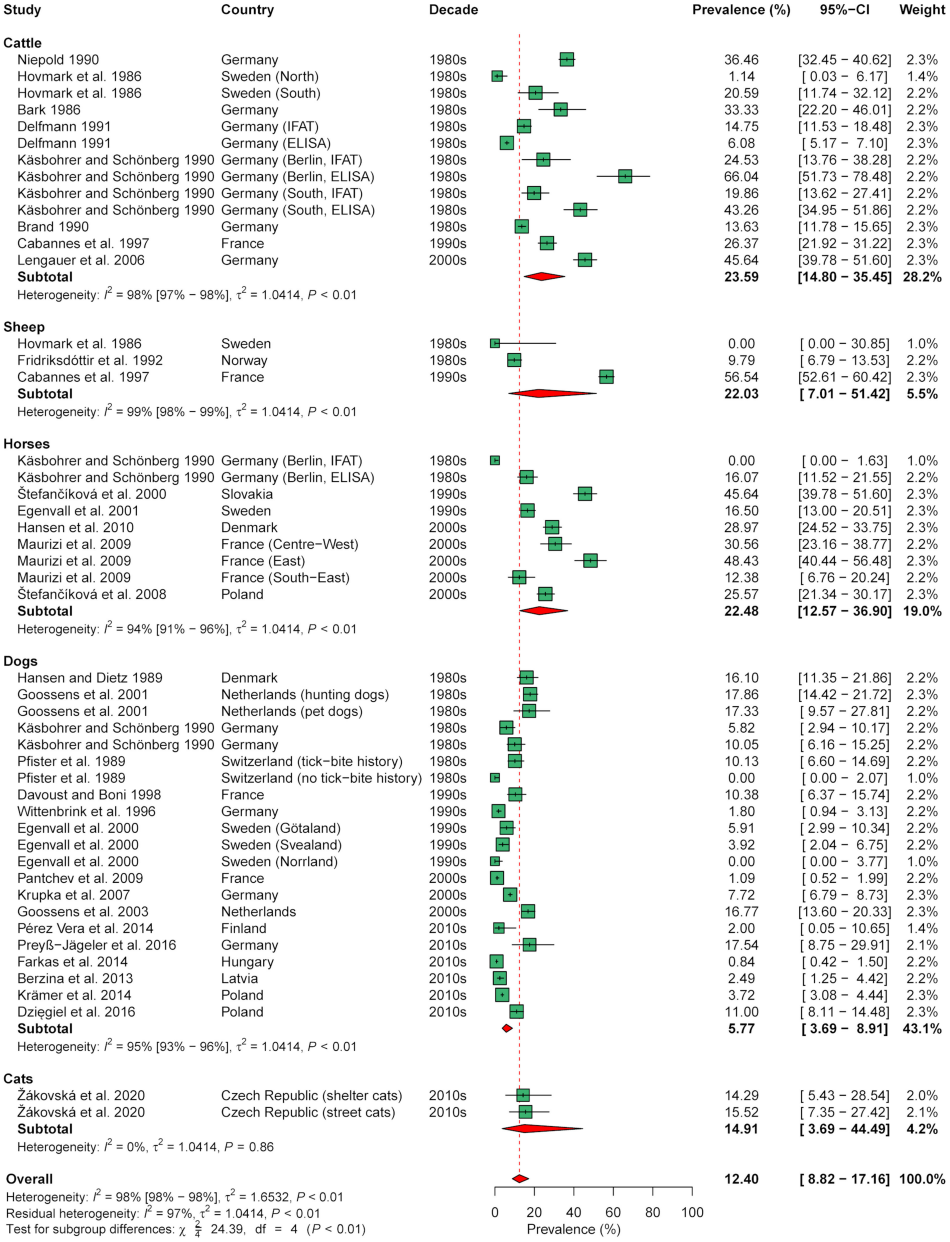
Forest plot displaying the results of random-effects meta-analysis of *B. burgdorferi* s.l. seroprevalence in domestic animals, with subgroup analysis according to animal species. Individual study results are shown as green squares, corresponding in size to the weight of the study on the overall prevalence estimate. Error bars indicate 95% confidence intervals (CI). Pooled prevalences are shown as red diamonds and the red dotted vertical line indicates the estimated overall prevalence.

Meta-analysis of seroprevalence, including 48 animal cohorts (13 cattle, 3 sheep, 9 horse, 21 dog, and 2 cat cohorts) from 30 publications, estimated an overall seroprevalence of 12.4% (95% CI: 0.1–17.2%) with a significant level of heterogeneity (*I*^2^ = 98.0%; 95% CI: 97.8–98.3%; *P* < 0.001). Subgroup analysis indicated a significant effect of animal species (χ^2^ = 24.4, df = 4, *P* < 0.001), with a lower seroprevalence in dogs (5.8%, 95% CI: 3.7–8.9%) than in cattle (23.6%, 95% CI: 14.8–35.4%) and horses (22.5%, 95% CI: 12.6–36.9%; Figure 5). In contrast, there were no significant differences according to decade (χ^2^ = 4.7, df = 3, *P* = 0.193), diagnostic test used (χ^2^ = 3.3, df = 2, *P* = 0.193), and geographical region (χ^2^ = 2.9, df = 2, *P* = 0.230) when analyzing all 48 cohorts.

Due to the significant effect of animal species, further analyses were conducted on the data subset for dogs, as this species had the largest sample size (21 cohorts from 16 studies). In the data subset on dogs, a significant effect of diagnostic test was found (χ^2^ = 7.5, df = 2, *P* = 0.023), with a lower seroprevalence determined by a C6-based rapid ELISA (3.1%, 95% CI: 1.5–6.0%) than by conventional ELISA (11.2%, 95% CI: 6.0–20.0%; Supplementary Figure 1). In contrast, no significant effect of geographical region (χ^2^ = 2.4, df = 2, *P* = 0.302) nor decade of sampling (χ^2^ = 3.5, df = 3, *P* = 0.324) was found in dogs.

#### Ruminants

Species-specific *Borrelia*-host associations are thought to be primarily driven by variation in resistance toward host defense mechanisms, particularly complement (59). Different species of the *B. burgdorferi* s.l. complex differ in their level of susceptibility toward inactivation of sera from certain animals *in vitro* [e.g., (60, 61)]. Notably, all tested members of the *B. burgdorferi* s.l. complex display high sensitivity toward serum complement from several ruminant species, including deer, bison, and cattle (59). Thus, these species seem to be irrelevant as *B. burgdorferi* s.l. reservoirs. In contrast, ticks feeding on these species may even lose their *Borrelia* infection, as suggested by prevalence patterns in engorged ticks recovered from deer, cattle and goats vs. prevalence in questing ticks (62, 63). Therefore, it has been suggested that an increase in grazing domestic ruminants may lower the risk of Lyme disease acquisition in a certain area (62). However, this does not apply to all ruminants, as several *B. burgdorferi* s.l. species are resistant toward serum of sheep and their wild relatives, the mouflon (59), and sheep may sustain natural *B. burgdorferi* s.l. cycles in the absence of other tick hosts (64).

Despite their apparent ability to eliminate *B. burgdorferi* s.l. spirochaetes, active *B. burgdorferi* sensu stricto (s.s.), and *B. afzelii* infections with associated symptoms (skin erythema, fever, acute lameness due to arthritis) have been described in cattle in rare cases (65, 66). Other studies draw a connection between serological evidence of *B. burgdorferi* s.l. infection and clinical signs such as lameness and swollen joints (67), but causality in these cases is extremely questionable. Experimental infections of cattle with *B. burgdorferi* s.s., *B. garinii*, and *B. afzelii* produced no clinical signs (68). Similarly, clinical manifestations of LB have been suspected in sheep (69), but experimental infections failed to produce any symptoms (70). Regarding goats, neither clinical cases nor infection experiments have been published to the authors' knowledge. Overall, clinical relevance of *B. burgdorferi* s.l. for ruminants is questionable. Nevertheless, they seroconvert upon contact with the pathogen (68) and may therefore be regarded as sentinels for pathogen presence.

In the present survey, 12 studies reporting seroprevalence rates in cattle were retrieved (six from Germany, one each from France, Poland, Slovakia, Sweden, and Switzerland and one reporting data from Poland as well as Slovakia; Supplementary Table 2). Reported seroprevalences ranged from 1.1% in northern Sweden (71) to 66.0% in Germany (72). Five studies reported data on sheep (two from Sweden, one each from France, Norway and Slovakia), with seroprevalences ranging from 0.0% in healthy sheep in central Sweden to 84.6% in lambs with arthritis on the island of Gotland, Sweden (71). The highest seroprevalence in asymptomatic sheep was determined in France (56.5%) (73). The only study containing data on goats reported a 17.2% seroprevalence rate in this species in Slovakia (74). Direct pathogen detection, e.g., by PCR, was not reported for ruminants in the considered studies.

#### Horses

In horses, a broad spectrum of clinical manifestations, including arthritis, lameness, anterior uveitis, encephalitis, and abortion, has been attributed to *B. burgdorferi* s.l. infection; however, in many cases a causal relationship has not been conclusively proven (75). Experimental inoculations of ponies led to systemic infection, persisting for at least 9 months, but did not induce any clinical signs nor histopathological alterations, except for skin lesions (76, 77). More recently, however, several case reports of equine neuroborreliosis with *B. burgdorferi* s.l. detection in the central nervous system have been published (78–81). In one of these studies, the species was identified as *B. burgdorferi* s.s. and high spirochaetal loads were demonstrated in tissues with inflammation (79). However, all of these cases occurred in North America, thus, it remains unclear if European *B. burgdorferi* s.s. isolates and other LB agents are capable of causing clinical manifestations in horses. Evidence from *in vitro* studies suggests that all tested *B. burgdorferi* s.l. species are susceptible to inactivation by equine complement, except for *B. burgdorferi* s.s. which displays an intermediate sensitivity (59).

In contrast to the paucity of conclusive clinical equine borreliosis cases in Europe, six studies evaluating seroprevalence rates in this species were retrieved (one each from Poland, Denmark, France, Germany, and Sweden and one reporting data from both Poland and Slovakia). Reported seroprevalence rates ranged from 12.4% in south-eastern to 48.4% in eastern France (82). Studies employing direct detection methods, e.g., PCR, were not retrieved.

#### Dogs and Cats

According to a consensus statement by the American College of Veterinary Internal Medicine (ACVIM), most *B. burgdorferi* s.l. seropositive dogs and cats display no clinical signs, neither after natural nor experimental infections (58). A small subset of dogs, however, may develop arthritis due to *B. burgdorferi* s.s. infection, as demonstrated by experimental infections (83–85). Furthermore, nephritis is putatively associated with *B. burgdorferi* s.s. infections, however, well-documented case reports are rare and no experimental studies exist in this regard (58). To the authors' knowledge, evidence for clinical manifestations due to other species of the *B. burgdorferi* s.l. complex in dogs is lacking to date, although *B. afzelii* has been isolated from a dog with clinical signs attributable to LB in Europe (86). Furthermore, DNA of *B. valaisiana* and *B. garinii* has been amplified from symptomatic dogs [(87, 88); see below]. In experimental studies, dogs have been shown to transmit borreliae to ticks, indicating a potential reservoir function (89). However, in the light of abundant wild reservoir hosts, such as rodents and birds, the impact of pet dogs on the natural epidemiological cycle is probably neglectable (90).

Cats have been infected experimentally and show seroconversion, but no clinical signs of infection (91, 92). Some case reports have attributed clinical signs in cats, such as cardiac arrhythmia and lameness, to *B. burgdorferi* s.l. infection based on seropositivity, PCR detection of the pathogen and/or resolution upon antibiotic treatment (93, 94); however, as in many other cases a causative relationship remains speculative.

Nevertheless, seroprevalence in domestic dogs and cats may provide an estimate of human LB risk. A strong association was found between canine seroprevalence and mean LB incidence on county level in the United States of America (95).

In the present survey, 38 studies reporting *B. burgdorferi* s.l. (sero-)prevalences in dogs were compiled (seven each from Germany and Poland, four from Sweden, three each from the Netherlands, Slovakia, and Switzerland, two each from the Czech Republic and France and one each from Austria, Denmark, Finland, Hungary, Latvia, Lithuania, and Norway). Seroprevalences ranging from 0.0% in northern Norway (96) to 57.5% in Bernese Mountain dogs in Switzerland (97) were reported. This dog breed seems to have a predisposition for *B. burgdorferi* s.l. infection, as demonstrated by several studies (97–99), although the reasons for this predisposition are unknown (58). As reported above, a significant effect of the utilized diagnostic test on the seroprevalence in dogs was found (section Tick-Borne Encephalitis Virus, Supplementary Figure 1), with lower rates determined by the most recently developed test, a C6-based rapid ELISA, than by conventional ELISA or IFAT (57).

Some studies also reported PCR detection rates in dogs, ranging from 0.0% in asymptomatic dogs (100) to 60.0% in animals with clinical signs attributable to borreliosis (87). DNA of *B. burgdorferi* s.s., *B. afzelii, B. valaisiana*, and *B. garinii* was amplified in these cases (87, 88).

Three studies reported data on cats (one each from the Czech Republic and the United Kingdom, one based on cats from different European countries). Two of these assessed seroprevalence, which ranged from 2.2% in symptomatic cats from different European countries, determined by a C6-based rapid ELISA (101), to 19.2% of cats presented at veterinary clinics in the Czech Republic, determined by conventional ELISA (102). The third study determined a 1.6% *B. burgdorferi* s.l. infection rate in systemically ill cats in the United Kingdom by PCR (103).

### Borrelia miyamotoi

*Borrelia miyamotoi* was first isolated from *Ixodes persulcatus* in Japan in 1995 (19), but pathogenicity for humans was not recognized until 2011 (20). Since then, this pathogen has been known to cause a febrile illness (104) and, more recently, it has been associated with meningoencephalitis in immunocompromised patients (105, 106). Similar to *B. burgdorferi* s.l., rodents and birds seem to be reservoir hosts for this spirochaete (104). Little is known about the role of domestic animals regarding *B. miyamotoi* ecology. In contrast to *B. burgdorferi* s.l., prevalence of *B. miyamotoi* in ticks is not negatively affected by the presence of cattle (62, 107). *Borrelia miyamotoi* DNA has been detected in two healthy cats in the USA (108). However, infection of other domestic animals has not been documented so far to the authors' knowledge and no (sero-)prevalence studies have been conducted.

### Anaplasma phagocytophilum

*Anaplasma phagocytophilum* is regarded as an important zoonotic pathogen, causing disease in humans, domestic ruminants, horses, dogs, and rarely in cats, while wild mammals act as reservoir hosts (109). This obligate intracellular rickettsial pathogen replicates in neutrophilic granulocytes and leads to thrombocytopenia, leukopenia, anemia, and immunosuppression associated with variable clinical signs (110). The epidemiology of *A. phagocytophilum* is complex due to the circulation of various strains and ecotypes, and shows considerable differences between Europe and North America (109). Human, equine, and canine granulocytic anaplasmosis have been described in Asia, Europe, and North America, while domestic ruminants only seem to be affected in Europe (109). Based on *groEL* genetic sequences, eight haplotype clusters of *A. phagocytophilum* were recently identified, and most isolates from humans and domestic animals belong to cluster 1, while other clusters, e.g., containing samples from roe deer, do not seem to be zoonotic (111).

**Figure 6:**
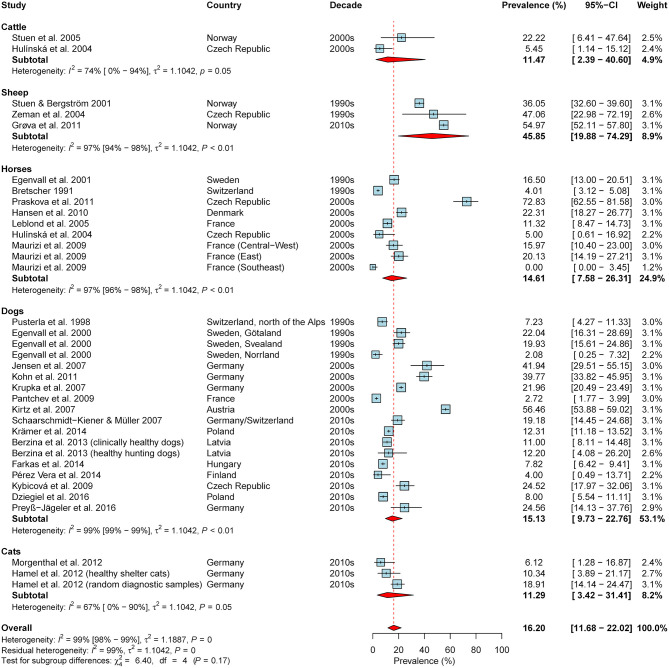
Forest plot displaying the results of random-effects meta-analysis of *A. phagocytophilum* seroprevalence in domestic animals, with subgroup analysis according to animal species. Individual study results are shown as blue squares, corresponding in size to the weight of the study on the overall prevalence estimate. Error bars indicate 95% confidence intervals (CI). Pooled prevalences are shown as red diamonds and the red dotted vertical line indicates the estimated overall prevalence.

Overall, 65 studies reporting (sero-)prevalence rates of *A. phagocytophilum* in domestic animals in the study region were retrieved (Supplementary Table 3). The meta-analysis based on seroprevalence rates of asymptomatic animals, including data on 35 animal cohorts (2x cattle, 3x sheep, 9x horses, 18x dogs, 3x cats) from 28 publications, yielded an estimated overall seroprevalence of 16.2% (95% CI: 11.7–22.0%), with a significant level of heterogeneity (*I*^2^ = 98.6%; 95% CI: 98.4–98.8%; *P* < 0.001). Although rather high seroprevalence rates were detected in sheep as compared to the other animal species, subgroup analyses indicated no significant difference (test for subgroup differences: χ^2^ = 6.4, df = 4, *P* = 0.171; Figure 6). Likewise, significant differences according to geographical region (χ^2^ = 0.38, df = 2, *P* = 0.828), the decades of sampling (χ^2^ = 0.78, df = 2, *P* = 0.676; Figure 6) or the type of diagnostic test were not detected (χ^2^ = 4.98, df = 2, *P* = 0.083).

#### Ruminants

In domestic ruminants, the disease caused by *A. phagocytophilum* is known as tick-borne fever and presents with fever, anorexia, abortion and a drop in milk production (109). In sheep but not in cattle, immunosuppression is also common, frequently resulting in secondary infections (112), which may be fatal in some cases (113). After recovery, sheep develop persistent infections with recurrent phases of high bacteraemia for at least 1 year, suggesting they may also act as a pathogen reservoir (114, 115). Furthermore, field data suggest that cattle can also become persistently infected or are frequently re-infected, indicating a possible reservoir function, which needs to be explored further (116). Recent genetic analyses have shown that *A. phagocytophilum* isolates from sheep and cattle in Europe cluster with isolates from humans, dogs, and horses (117).

In total, six studies reported (sero-)prevalence rates for cattle (two studies from Sweden and one study each from Belgium, the Czech Republic, Norway, and Switzerland), seven for sheep (two each from the Czech Republic and Norway, one each from Denmark, Germany and Sweden), and two for goats (Switzerland and United Kingdom, Supplementary Table 3).

In cattle, seroprevalence rates varied from 5.5% in the Czech Republic (118) to 100% in a clinically-affected herd in Norway (119). The highest published seroprevalence rate in asymptomatic cattle was 63.0% in a Swiss study (120). Three studies reported prevalence rates based on PCR, which ranged from 5.5% in the Czech Republic (118) to 85.7% in symptomatic animals in Sweden (121).

In sheep, prevalence of *A. phagocytophilum* antibodies ranged from 36.0% in Norway (122) to 100% in a flock in the Czech Republic (123). PCR-determined prevalence rates in sheep varied from 2.9% in the Czech Republic/Slovakia (124) to 41.9% in sheep flocks with high lamb morbidity and mortality in Sweden (43). Regarding goats, a PCR-determined prevalence of 5.6% was reported from Switzerland (125). In addition, four of five feral goats caught in Northern Ireland, UK, were PCR-positive (126).

However, (sero-)prevalence rates in ruminants may be difficult to compare between studies, since marked seasonal variation has been found. For example, in a Swiss study, seroprevalence of two cattle herds varied between 16% before and 63% at the end of the grazing season (120). As most ruminants are housed during winter, determined (sero-)prevalence rates greatly depend upon the season of sampling.

In addition, serologic cross-reactivity with other *Anaplasma* spp., e.g., *Anaplasma marginale* in cattle (127) and probably also *Anaplasma ovis* in sheep (128), needs to be considered. In Europe, *A. marginale* occurs mainly in the Mediterranean region, but also in Switzerland, Austria, and Hungary (129). *Anaplasma ovis* has been detected in France (130) as well as Slovakia (124) and Hungary (129).

#### Horses

In horses, *A. phagocytophilum* may cause an acute febrile disease with depression, anorexia, ataxia, icterus, and lower limb oedema, which is usually self-limiting (131). Similar to sheep, horses may develop persistent subclinical infections with recurrent bacteraemia after spontaneous recovery from acute disease (132). Notably, equine *A. phagocytophilum* strains seem to be similar or identical to those causing disease in humans and dogs (133).

Most equine granulocytic anaplasmosis (EGA) cases have been reported from European countries (131). In the present investigation, 11 studies reporting (sero-)prevalence rates of *A. phagocytophilum* in horses were identified, two each from the Czech Republic, France, and Sweden and one each from Denmark, France, the Netherlands, Switzerland, and Sweden. Another two studies reported data from Poland, Slovakia and the Ukraine and from Germany, Poland and the Ukraine, respectively. Reported seroprevalence rates ranged from 4.0% in Switzerland (134) to 72.8% in the Czech Republic (135). Remarkably, the latter study was based on healthy horses. Prevalences determined by PCR ranged from 0.0% in the Ukraine (136) to 62.9% in Sweden, whereby the latter study was based on horses presenting symptoms attributable to EGA.

#### Dogs and Cats

Canine granulocytic anaplasmosis (CGA) is regarded as one of the most important vector-borne diseases in Europe. While many cases are probably subclinical, acute febrile illness may also occur (137). Frequent clinical signs are lethargy, anorexia, and pale mucous membranes, sometimes accompanied by enlarged lymph nodes, bleeding (petechias, epistaxis), and immune-mediated arthritis (137). Similar symptoms may be seen in cats infected with *A. phagocytophilum*, although experimental studies indicate that symptoms are usually mild (138). Experimental infections have been shown to persist for at least 5^1^/_2_ months in dogs (139) and 3 months in cats (91). The *A. phagocytophilum* strains causing disease in dogs seem to be zoonotic (117).

In total, 37 studies on *A. phagocytophilum* (sero-)prevalence in dogs were retrieved from the literature, including 10 from Germany, six from Poland, five from Sweden, two each from Austria, the Czech Republic, Hungary Slovakia, and Switzerland, and one study each from Finland, France, Latvia, Lithuania, and the United Kingdom. One study included dogs from Germany and Switzerland. Reported seroprevalence rates, determined either by IFAT or (rapid) ELISA, ranged from 2.1% in northern Norway (96) to 56.5% in Austria (140) (Supplementary Table 3). However, the latter study included dogs with symptoms possibly related to CGA. Since infection may not lead to clinical disease, and CGA is characterized by rather unspecific symptoms, several studies did not find a significant difference in seroprevalence between apparently healthy animals and those presenting some form of illness [e.g., (100, 141)]. Nevertheless, cohorts of symptomatic dogs were excluded from the meta-analysis on seroprevalence. Serologic tests available for assessing *A. phagocytophilum* exposure may cross-react with *Anaplasma platys* antibodies, therefore, seroprevalence may be overestimated. However, as *A. platys* transmission in Europe is restricted to Mediterranean countries (137), this pathogen only plays a role as an imported bacterium in the countries considered in this review, with the exception of southern France. Nevertheless, with increased travel activity and import of dogs by animal welfare organizations, this should be kept in mind when interpreting seroprevalence rates.

Prevalence rates determined by PCR ranged from 0.0% in Switzerland and Poland (142, 143) to 66.7% in dogs with symptoms attributable to CGA in Sweden (121). The highest prevalence in apparently healthy dogs was 12.2% in a group of Latvian hunting dogs (144).

Regarding cats, six studies were found (four from Germany, one from the United Kingdom and one from Ireland). These studies reported IFAT-determined seroprevalence rates from 6.1% in healthy cats (145) to 18.9% in random diagnostic samples (146). PCR detection rates varied from 0.0% in healthy cats (145) to 4.3% in necropsy samples from shelter cats (147).

### Neoehrlichia mikurensis

*Neoehrlichia mikurensis*, a member of the family Anaplasmataceae, was first discovered in the early 2000s, recognized as a human pathogen in 2010 and recently cultivated in tick cell lines as well as human endothelial cells (148). It occurs in *I. ricinus* populations throughout Europe and human cases, mainly involving immunosuppressed patients, have been reported from several countries (149). Regarding domestic animals, knowledge on the relevance of *N. mikurensis* as a pathogenic agent and respective prevalence data are scarce.

In the present literature survey, five studies investigating *N. mikurensis* occurrence in domestic animals (four in dogs and one in cats) by PCR, but no seroprevalence studies, were obtained (Table 1). Infections in dogs seem to be rare, as only 0.3% of 1,023 dogs in Germany (151) and 0.1% of 889 dogs in Switzerland were infected (142). The positive dog in Switzerland was splenectomised (142). Another positive dog died of haemolytic anemia in the Czech Republic (150) and in a case report from Germany, canine *N. mikurensis* infection was associated with neutropenia and thrombocytopenia (23). However, the clinical relevance of *N. mikurensis* in dogs is still unclear (23, 142). A single study also tested spleen samples from 141 cats in Germany, but *N. mikurensis* was not detected (147). Other domestic animal species have not been investigated so far to the authors' knowledge.

**Table 1:** (Sero-)prevalence studies on *Neoehrlichia mikurensis* in domestic animals in temperate and cold regions of Europe.

**Country**	**Region**	**Year(s) of sampling**	**Method(s)**	**Positive/total**	**Prevalence**	**Comment(s)**	**References**
**DOGS**							
Czech Republic	NA	2009–2012	PCR	1/19	5.3%	Dogs with fatal immunhaemolytic anemia	(150)
Germany	Brandenburg	2013–2014	HRM PCR	3/1,023	0.3%		(151)
Hungary	Somogy	NA	PCR	0/90	0.0%	*Candidatus* Neoehrlichia lotoris-like detected in 6 dogs	(152)
Switzerland	Zurich	2005–2006	Real-time PCR	1/889	0.1%	The positive dog was splenectomised	(142)
**CATS**							
Germany	Berlin	2006–2008	HRM PCR	0/141	0.0%	Spleen samples from shelter cats	(147)

*HRM PCR, high-resolution melt PCR*.

### *Rickettsia* spp.

Several tick-transmitted human-pathogenic *Rickettsia* spp. occur in Europe. While the causative agent of Mediterranean spotted fever, *Rickettsia conorii*, has been known since the beginning of the twentieth century, several further *Rickettsia* spp. and their associated syndromes were described in the 1990s and 2000s (153). In central and northern Europe, *R. helvetica*, transmitted by *I. ricinus*, is probably the most frequent species. It causes a mild febrile illness in humans and is only sometimes associated with skin rash (154). In addition, *I. ricinus* may transmit *Rickettsia monacensis*, which leads to a clinical picture similar to Mediterranean spotted fever (153). *Rickettsia slovaca* and *R. raoultii*, causative agents of scalp eschar and neck lymphadenopathy (SENLAT), are transmitted by *Dermacentor* species (153).

With the exception of dogs, domestic animals do not seem to be susceptible to disease caused by human-pathogenic *Rickettsia* species. In dogs, infection with *Rickettsia rickettsii*, which causes Rocky Mountain spotted fever in North America, leads to clinical signs similar to those in humans (155). In addition, *R. conorii* has been associated with canine febrile illness (156). Dogs are also capable of transmitting *R. conorii* to ticks and may thus exert a reservoir function (157). However, canine disease due to the *Rickettsia* spp. relevant in central and northern Europe or a respective reservoir function have not been reported to the authors' knowledge.

Overall, domestic animals can mainly be regarded as sentinels for human exposure to *Rickettsia* spp. in central and northern Europe. However, only nine studies were identified (three from Germany, two from Switzerland, one each from the Czech Republic, Ireland, Poland, and Sweden), reporting data on horses, dogs, and cats (Table 2). Regarding domestic ruminants, no studies on *Rickettsia* (sero-)prevalence in the considered geographical region were obtained. The only study on horses reported a 36.5% *R. helvetica* seroprevalence in Sweden (158).

**Table 2:** (Sero-)prevalence studies on tick-transmitted *Rickettsia* spp. in domestic animals in temperate and cold regions of Europe.

**Country**	**Region**	**Year(s) of sampling**	**Method(s)**	**Positive/total**	**Prevalence**	**Comment(s)**	**References**
**HORSES**							
Sweden	NA	2010–2011	IFAT	23/63	36.5%	*R. helvetica* used as antigen	(158)
**DOGS**							
Czech Republic	NA	2009–2012	PCR	0/19	0.0%	Dogs with fatal immunhaemolytic anemia	(150)
Germany	Nationwide	2012–2014	ELISA^a^	469/602	77.9%	Dogs that never left Germany	(159)
	Nationwide	2012–2014	Micro-IFAT	568/605	93.9%	Same samples as in (159); clearly differentiable samples: 66.0% *R. helvetica*, 2.8% *R. raoultii*, 1.6% *R. slovaca*	(160)
	Brandenburg	2013–2014	PCR	8/1,021	0.8%	Identified species: 7x *R. raoultii*, 1x *R. felis*	(151)
Poland	North-Western Poland	NA	PCR	0/100 (group 1), 0/92 (group 2), 0/50 (group 3)	0.0% (group 1), 0.0% (group 2), 0.0% (group 3)	Group 1: healthy shelter dogs, group 2: suspected borreliosis, group 3: diagnosed babesiosis	(161)
Sweden	NA	2010–2011	IFAT	17/100	17.0%	*R. helvetica* used as antigen	(158)
Switzerland	Zurich	2005–2006	Real-time PCR^b^	0/889	0.0%		(142)
	Zurich	NA	Real-time PCR^b^	0/884	0.0%		(162)
**CATS**							
Ireland	Dublin	2008	PCR	0/121	0.0%		(163)
Sweden	NA	2010–2011	IFAT	19/90	22.1%	*R. helvetica* used as antigen	(158)

a *Commercially available, detects all spotted-fever group rickettsiae*.

b *Specific for R. helvetica*.

*ELISA, enzyme-linked immunosorbent assay; IFAT, immunofluorescence antibody test*.

In dogs, a high level of exposure to spotted-fever group rickettsiae was reported, with seroprevalence rates ranging from 17.0 to 93.9% (158, 160). When *R. helvetica*-specific antigens were used, seroprevalences of 17.0% (158) and 66.0% (160) were determined. Despite this high level of exposure, *Rickettsia* DNA (mainly *R. raoultii*) was found in only 0.8% of tested dogs in Germany (151), whereas two PCR-based studies from Switzerland (142, 162) and one study from Poland (161) reported 0.0% prevalence. Similarly, *R. helvetica* antibodies were detected in 22.1% of tested cats in Sweden (158), but no *Rickettsia* DNA was amplified from 121 tested cats in Ireland (163). Therefore, it seems unlikely that dogs and cats contribute to the epidemiology of tick-transmitted rickettsioses as reservoir hosts in northern and central Europe.

### Zoonotic *Babesia* spp.

Piroplasms of the genus *Babesia* are tick-transmitted protozoan parasites, which usually display a high degree of host specificity. Nevertheless, a few species are zoonotic, predominantly affecting immunocompromised patients (24). In Europe, most human infections are caused by the cattle parasite *B. divergens* (154). On the American continent, human babesiosis due to *Babesia microti*, which is rodent-associated, is more common. *Babesia microti* also occurs in Europe. However, clinically symptomatic human *B. microti* infections reported in Europe were mostly acquired in the Americas, so it is unclear whether European *B. microti* strains are human-pathogenic (154). Furthermore, *Babesia venatorum*, a parasite of deer, has been recognized as a human pathogen in immunocompromised patients in Europe (154).

Regarding domestic animals, only cattle are affected by and act as reservoirs for a *Babesia* spp. with zoonotic relevance, namely *B. divergens*, whereas the species parasitizing horses, sheep, goats, and dogs are not zoonotic. No cat-specific *Babesia* species are distributed in Europe. *Babesia microti* DNA has been detected in cats in southern Europe (e.g., in Italy) but the relevance of this finding remains unclear (164).

In cattle, *B. divergens* infection may lead to severe haemolytic anemia, which can be fatal (165, 166). Symptoms consist of fever, pale or jaundiced mucous membranes, anorexia, weakness, elevated heart and respiratory rates and hemoglobinuria, hence the colloquial name of the disease, “redwater” (165). Recovering animals acquire immunity, which is maintained by repeated pathogen exposure (165). Calves under the age of ~9 months display higher resistance toward clinical disease and are subsequently immunologically protected (167, 168), thus, clinical disease in endemic situations usually only occurs in immunologically naïve animals, which were either recently introduced to the area or had no access to pasture during the first year of life (165).

In the present survey, 18 studies reporting *Babesia* (sero-)prevalence data in cattle were retrieved (four each from France, Germany, and the United Kingdom, two from Belgium, and one each from Hungary, Norway, Sweden, and Switzerland; Table 3). Reported seroprevalence rates, determined mostly by IFAT, ranged from 0.0% in asymptomatic animals in Northern Germany (177) to 100% in animals presenting with acute babesiosis in France (172). The highest seroprevalence in randomly chosen individuals, but from a region where babesiosis was known to occur, was 90.6% at the end of the grazing season (167). The latter study also showed that seroprevalence varied throughout the year, similar to the pattern described above for *A. phagocytophilum* (120), making different studies difficult to compare. Keeping this draw-back in mind, an overall seroprevalence of 7.4% (95% CI: 2.6–19.2%) was estimated based on 11 healthy or randomly chosen cattle cohorts (Supplementary Figure 2). A significant level of heterogeneity was detected (*I*^2^ = 99.6%; 95% CI: 99.6–99.7%; *P* < 0.001). No significant temporal change in seroprevalence was detected from the 1970s to the 2000s (test for subgroup differences, χ^2^ = 0.96, df = 2, *P* = 0.619), although a decline in bovine babesiosis prevalence and/or clinical incidence has been postulated for several European countries (179, 180, 186). This may be due to the fact that several studies reporting this decline were based on clinical incidence (179, 186) and were thus not included in the calculation. Furthermore, no difference according to geographical region was found (χ^2^ = 5.8, df = 3, *P* = 0.121).

**Table 3:** (Sero-)prevalence studies on zoonotic *Babesia* spp. in domestic animals in temperate and cold regions of Europe.

**Country**	**Region**	**Year(s) of sampling**	**Method(s)**	**Positive/total**	**Prevalence**	**Comment(s)**	**Referencea**
**Cattle (*****B. divergens*****)**							
Belgium	Central Belgium	1988	IFAT	136/1,721	7.9%		(169)^*^
	Southern Belgium	2010	IFAT	7/65 (spring), 13/65 (summer), 8/65 (autumn)	10.7% (spring), 20.0% (summer), 12.3% (autumn)	Farms with a known history of babesiosis or anaplasmosis	(170)
France	Nationwide	1988	Blood smears / inoculation in gerbils	374/424	88.2%	Animals treated for clinical babesiosis	(171)
	Sarthe	1991	ELISA	115/200	57.5%	Farms with clinical babesiosis during the last 5 years	(171)
	Ille-et-Vilaine	2001–2002	IFAT, *in vitro* culture	19/19 (IFAT, group 1), 31/77 (IFAT, group 2), 19/19 (culture, group 1), 31/77 (culture, group 2)	100% (IFAT, group 1), 40.3% (IFAT, group 2), 100% (culture, group 1), 40.3% (culture, group 2)	Group 1: acute babesiosis, group 2: asymptomatic	(172)^*^
	Mid-eastern France	2001–2002	IFAT, PCR	18/254 (IFAT), 12/254 (PCR)	7.1% (IFAT), 4.7% (PCR)		(173)^*^
	Western France	2007	IFAT	102/711	14.3%		(174)^*^
Germany	Bavaria	1982	IFAT, ELISA	211/1,616	13.1%		(175)^*^
	Northern Germany	1984–1985	IFAT	108/251	43.0%	Farms with history of babesiosis, includes vaccinated animals	(176)
	Northern Germany	1988–1990	IFAT	0/212 (group 1), 0/354 (group 2), 8/200 (group 3)	0.0% (group 1), 0.0% (group 2), 4.0% (group 3)	Group 1: *Borrelia*-positive animals, group 2: farms with suspected babesiosis, group 3: farms with history of babesiosis	(177)^*^
	Bavaria	2002	IFAT	1/287	0.4%		(178)^*^
Hungary	Northeastern Hungary	2005	IFAT	2/654	0.3%		(179)^*^
Norway	Southern Norway	2004–2005	IFAT	84/306	27.4%		(180)^*^
Sweden	Southern Sweden	NA	Real-time PCR	38/71	53.5%	Includes 39 cattle with symptoms of babesiosis	(181)
Switzerland	Jura Canton	1981	IFAT	98/289 (April), 190/327 (July), 309/341 (December)	33.9% (April), 58.1% (July), 90.6% (December)		(167)
United Kingdom	Scotland	1976	IFAT	290/368	78.8%	Two farms which experienced babesiosis outbreak	(182)
	Scotland	NA	IFAT	2,522/22,044	11.4%		(183)^*^
	Northern Ireland	1978	IFAT	5,731/18,000	31.8%		(184)^*^
	Scotland	2014	PCR	0/107	0.0%	Farms with a known history of babesiosis or anaplasmosis	(185)
**SHEEP**							
United Kingdom	Scotland	2014	PCR	11/93	11.8%	Identified as *B. venatorum*	(185)

* *Included in meta-analysis of seroprevalence*.

*ELISA, enzyme-linked immunosorbent assay, IFAT, immunfluorescence antibody test*.

A few studies also reported detection of *B. divergens* by PCR, with infection rates ranging from 0.0% in Scotland (185) to 53.5% among clinically symptomatic cattle in Sweden (181). In randomly selected cattle in France, an infection rate of 4.7% was determined by PCR (173).

In addition to cattle, sheep might act as reservoirs for zoonotic *Babesia* spp., as *B. venatorum* infections have recently been detected in 11.8% of 93 studied sheep in the United Kingdom, whereas the 107 tested cattle were negative (185).

## Discussion

The present survey aimed at presenting an overview of the most important zoonotic TBDs in domestic animals in temperate and cold regions of Europe, where *I. ricinus* is the predominant tick vector. While a rather large number of studies on *B. burgdorferi* s.l., *A. phagocytophilum* and TBEV were retrieved, studies on *Rickettsia* spp. in domestic animals were few, probably because *Rickettsia* spp. have not (yet) been associated with clinical disease in these species. Therefore, the prevalence and clinical relevance of *Rickettsia* spp. in domestic animals represent a knowledge gap. Similarly, only few studies exist on the relatively recently discovered pathogen *N. mikurensis* in domestic animals, although first reports indicate that this pathogen may be of clinical relevance for dogs (150). Studies on relevant zoonotic *Babesia* spp. were only found with regard to *B. divergens* in cattle, while only one study investigated *B. venatorum*, which is usually deer-associated, in cattle and sheep. As *Babesia* spp. are characterized by a high level of host-specificity (187), it was not surprising that no prevalence studies were conducted on rodent-associated *B. microti* in domestic animals in the considered regions.

In general, a high level of heterogeneity was detected in the datasets. To limit this heterogeneity in meta-analysis of seroprevalence, only animal cohorts asymptomatic for the considered pathogen or random diagnostic samples were included, and further restrictions were applied regarding the diagnostic test used for TBEV in all domestic animals and *B. burgdorferi* s.l. in dogs. Nevertheless, heterogeneity remained high, even after conducting subgroup analyses according to species, diagnostic test (if applicable), geographic region and decade. A significant effect was only found regarding species differences in *B. burgdorferi* s.l. infections, with higher rates in domestic ruminants and horses than in dogs. On the one hand, this might be due to the close relationship between dogs and their owners, resulting in better protection from TBDs due to treatment with (repellent) acaricides, and shorter duration of tick attachment, as ticks are probably noticed sooner on dogs than on horses or cattle. On the other hand, the choice of diagnostic test may have contributed to the significantly lower *B. burgdorferi* s.l. seroprevalence in dogs. Although no significant effect of diagnostic test was found in the overall dataset, analyzing the data subset on dogs alone revealed a significant effect, with lower rates determined by the most recently developed test, a rapid ELISA with a high sensitivity and specificity for antibodies against the *Borrelia* C6 antigen (57). This test was almost exclusively used in dogs, except for two studies on horses. This indicates that cross-reactivity with other pathogens, e.g., *Leptospira* spp. (74), may have been an issue, especially in older and in non-canine studies.

Regarding *A. phagocytophilum*, conspicuously high seroprevalence rates in sheep were reported. However, as the meta-analysis included only three studies on sheep, no significant species differences were found when conducting subgroup analysis. However, this should be investigated further, as granulocytic anaplasmosis is a severe, possibly fatal disease in sheep (113). In addition, sheep might constitute a reservoir for human-pathogenic *A. phagocytophilum* strains (114, 115, 117). Thus, more studies on *A. phagocytophilum* (sero-)prevalence in sheep seem warranted.

In addition to species differences and different diagnostic tests used, the chosen cut-off level to determine seropositivity, the source of the antigen in serological tests or the timing of sampling—as seroprevalences may increase during seasons of tick exposure (120, 171)—may have further increased heterogeneity of study results. These aspects, hampering the comparability of studies, may have contributed to the fact that no temporal changes in TBD seroprevalence in domestic animals were found. Possibly, a larger number of studies may have been necessary to detect a temporal trend under these conditions. Furthermore, there were large temporal gaps in the available seroprevalence studies. For example, studies on TBEV considered suitable for meta-analysis were mainly conducted as of the year 2000, with only one comparable study from the 1960s and none from the decades in between. Regarding *Babesia* spp., there was a gap in available screening studies on asymptomatic animals concerning the 1990s as well as the 2010s. Regarding *B. burgdorferi* s.l., most studies were conducted in the 1980s, following the initial description of the pathogen (188).

In consequence, the trend of increasing TBD incidence in humans was not reflected by the available data from domestic animals. Apart from the mentioned drawbacks that may have masked such a trend, it is also possible that seroprevalences in domestic animals are rather stable and that the increased TBD incidence in humans is due to factors which are not relevant for animals. For example, increased exposure due to more outdoor activities or increased awareness of patients and physicians for TBDs may lead to an increased disease incidence or more diagnoses of TBD infections in humans (2).

With regard to the geographical spread of zoonotic TBDs, e.g., concerning TBE, (sero-)prevalence in animals is usually not studied in a certain area until there is an indication of pathogen presence due to human cases. Therefore, it is difficult to ascertain when the pathogen emerged in animal populations in this area. As an example, no studies on TBEV in domestic animals in the United Kingdom were retrieved, where TBEV has only recently been detected (17).

## Conclusions

This survey revealed a high heterogeneity in (sero-)prevalences of zoonotic TBDs in domestic animals in temperate and cold regions of Europe. In addition, temporal gaps in available studies were detected, e.g., for TBEV and *B. divergens*. The high level of heterogeneity as well as the temporal gaps make it difficult to assess long-term temporal trends for comparison with data on humans. Furthermore, only few studies were retrieved regarding *Rickettsia* spp. and the recently described pathogen *N. mikurensis*, and none regarding *B. miyamotoi*. Therefore, more studies investigating these neglected pathogens are warranted. Additionally, further surveillance studies employing highly sensitive and specific test methods and including hitherto non-investigated regions are needed to determine if and how a changing world impacts the frequency of neglected zoonotic tick-borne pathogens in domestic animals.

## Author Contributions

CS designed the study. AS, AG, and A-KT conducted the literature search. AS conducted statistical analyses and drafted the manuscript. All authors participated in data interpretation, reviewed the manuscript draft, read, and approved the final manuscript.

## Conflict of Interest

The authors declare that the research was conducted in the absence of any commercial or financial relationships that could be construed as a potential conflict of interest.
