# Supplementary material for: Zoonotic Tick-Borne Pathogens in Temperate and Cold Regions of Europe—A Review on the Prevalence in Domestic Animals
Source: Front Vet Sci. 2020 Dec 10;7:604910. doi: 10.3389/fvets.2020.604910 (PMC7758354; doi:10.3389/fvets.2020.604910)
Supplement: Supplementary file 1 [file Table_1.DOCX]

**Supplementary Table 1:** (Sero-)prevalence studies on tick-borne encephalitis in domestic animals in temperate and cold regions of Europe. Abbreviations: ELISA: enzyme-linked immunosorbent assay; HAI: haemagglutination inhibition test; RT-PCR: reverse transcription polymerase chain reaction; SNT: seroneutralization test

| Country | Region | Year(s) of sampling | Method(s) | Positive/total | Prevalence | Comment(s) | Reference^a^ |
| --- | --- | --- | --- | --- | --- | --- | --- |
| Cattle | | | | | | | |
| Belgium | Eastern Belgium | 2010 | SNT | 17/650 | 2.6% |  | (1)* |
| Finland | Nationwide | 1960-1963 | SNT+HAI | 13/6585 | 0.2% |  | (2)* |
| Hungary | Northern Hungary | 2005 | ELISA+SNT | 69/260 | 26.5% |  | (3)* |
| Lithuania | Nationwide | 2001 | HAI | 10/423 | 2.4% |  | (4) |
| Netherlands | Different parts of the country | NA | ELISA+SNT | 0/180 | 0.0% |  | (5)* |
| Norway | Coastal areas | NA | HAI | 14/81 | 17.3% |  | (6) |
|  | Different regions | 2014-2017 | ELISA+SNT (serum), real time RT-PCR (milk) | 15/112 (serum), 6/112 (milk) | 13.4% (serum), 5.4% (milk) |  | (7)* |
| Poland | Lublin | NA | ELISA | 5/123 | 4.1% |  | (8) |
| Sheep | | | | | | | |
| Germany | Bavaria | 2003 | ELISA+SNT | 48/1469 | 3.3% |  | (9)* |
|  | Bavaria | 2008-2009 | ELISA+SNT | 79/759 | 10.4% |  | (9)* |
|  | Baden-Wuerttemberg | 2008-2009 | ELISA+SNT | 25/399 | 6.3% |  | (9)* |
|  | Thuringia | 2008-2009 | ELISA+SNT | 58/452 | 12.8% |  | (9)* |
|  | North Rhine-Westphalia | 2008-2009 | ELISA+SNT | 0/119 | 0.0% |  | (9)* |
|  | Lower Saxony | 2008-2009 | ELISA+SNT | 2/177 | 1.1% |  | (9)* |
|  | Schleswig-Holstein | 2008-2009 | ELISA+SNT | 1/31 | 3.2% |  | (9)* |
|  | Mecklenburg Western-Pomerania | 2008-2009 | ELISA+SNT | 0/184 | 0.0% |  | (9)* |
|  | Mecklenburg Western-Pomerania | 2014-2017 | ELISA+SNT | 2/375 | 0.5% |  | (10)* |
| Hungary | Northern Hungary | 2005 | ELISA+WB+SNT | 7/100 | 7.0% |  | (3)* |
| Lithuania | Nationwide | 2001 | HAI | 5/118 | 4.2% |  | (4) |
| Slovakia | Eastern Slovakia | 1982-1983 | HAI | 20/755 | 2.6% |  | (11) |
| Sweden | Östergötland, Gotland | 2013 | ELISA | 11/43 | 25.6% | Farms with high lamb morbidity and mortality | (12) |
|  | Örebro | 2018 | ELISA+SNT | 19/247 | 7.7% | Milk and colostrum samples | (13)* |
| Goats | | | | | | | |
| Austria | Alpine region | 2008 | HAI+SNT | 1/106 | 0.9% | Goat caused an alimentary TBEV outbreak | (14)* |
| Germany | Baden-Wuerttemberg | 2006-2009 | ELISA+SNT | 102/2240 | 4.6% |  | (9)* |
|  | Baden-Wuerttemberg | 2013-2015 | ELISA+SNT | 7/174 | 4.0% |  | (15)* |
|  | Bavaria | 2003/2009 | ELISA+SNT | 3/725 | 0.4% |  | (9)* |
|  | Bavaria | 2013-2015 | ELISA+SNT | 1/230 | 0.4% |  | (15)* |
|  | Baden-Wuerttemberg, Thuringia | 2010-2011 | ELISA+SNT | 13/100 | 13.0% | Same flocks, but not the same animals, as in (9) | (16)* |
|  | Thuringia | 2009 | ELISA+SNT | 16/828 | 1.9% |  | (9)* |
|  | Lower Saxony | 2013-2014 | ELISA+SNT | 1/126 | 0.8% |  | (15)* |
|  | Mecklenburg-Western Pomerania | 2013 | ELISA+SNT | 1/205 | 0.5% |  | (15)* |
|  | Mecklenburg-Western Pomerania | 2014-2017 | ELISA+SNT | 0/104 | 0.0% |  | (10)* |
| Lithuania | Nationwide | 2001 | HAI | 4/561 | 0.7% |  | (4) |
| Poland | Different regions | 2002-2006 | ELISA | 17/358 | 4.7% |  | (17) |
| Sweden | Örebro | 2018 | ELISA+SNT | 0/17 | 0.0% |  | (13)* |
| Switzerland | Valais | 2011-2012 | ELISA+SNT | 70/4114 | 4.2% | All goats > 6 months of age | (18)* |
|  | Ticino | 2014-2016 | ELISA+SNT | 97/662 | 14.6% |  | (19)* |
| Horses | | | | | | | |
| Austria | Nationwide | 1995-1998 | ELISA+SNT | 60/469 | 12.8% | Includes animals with neurologic symptoms | (20) |
|  | Different regions | 2011 | ELISA+SNT | 67/257 | 26.1% |  | (21)* |
| Germany | Thuringia | 2009 | ELISA+SNT | 1/130 | 0.8% | Mostly herds from non-TBE risk areas | (22)* |
|  | Bavaria | 1999-2000 | ELISA (+SNT) | 48/205 | 23.4% | Only a subset of samples confirmed by SNT | (23) |
|  | Hesse | 2003 | SNT | 7/240 | 2.9% |  | (24)* |
|  | Bavaria | 2011 | ELISA+SNT | 2/10 (herd 1),  5/15 (herd 2) | 20.0% (herd 1), 33.0% (herd 2) | Two herds from TBE risk areas | (22)* |
| Hungary | Northern Hungary | 2005 | ELISA+SNT | 0/40 | 0.0% |  | (3)* |
| Slovakia | Different regions | 2013 | ELISA+SNT | 5/145 | 3.4% | Horses with and without travel history | (25)* |
| Dogs | | | | | | | |
| Different European countries^b^ |  | 2013-2015 | ELISA | 47/408 | 11.5% | Dogs with neurological illness | (26) |
| Austria | Nationwide | 1996-1998 | ELISA+SNT | 110/545 | 20.2% | Includes dogs with neurological illness | (27) |
| Belgium | Nationwide | 2009 | ELISA+SNT | 1/880 | 0.1% | Positive dog with travel history to an endemic area | (28)* |
| Czech Republic | Moravia, Bohemia | 1997-1998 | HAI | 5/151 | 3.3% | Includes dogs with neurological illness | (29) |
|  | South Moravia | 2011-2012 | cELISA, real-time RT-PCR | 18/159 (cELISA), 20/159 (PCR) | 11.3% (cELISA), 12.6% (PCR) | Includes dogs with neurological illness | (30) |
| Denmark | Different regions | 2005-2006 | ELISA+SNT | 6/125 | 4.8% | Only dogs without travel history to TBEV-endemic areas outside Denmark | (31)* |
| Finland | Different regions | 2011-2012 | ELISA+IFAT | 9/148 | 6.1% |  | (32) |
| Germany | Bavaria | 2000 | ELISA (+SNT) | 71/243 | 29.2% | Only a subset of samples confirmed by SNT | (23) |
|  | Saxony | 2012-2014 | ELISA+SNT | 7/331 | 2.1% | Only dogs without travel history | (33)* |
|  | NA | NA | ELISA | 17/54 (group 1), 30/56 (group 2) | 30.4% (group 1),  53.6% (group 2) | Group 1: healthy dogs, group 2: dogs with neurological symptoms | (34) |
| Netherlands | Different parts of the country | NA | ELISA+SNT | 0/24 | 0.0% |  | (5)* |
| Norway | Southern Norway | 1992-2000 | Two different ELISAs | 52/317 | 16.4% |  | (35) |
| Poland | Mazowiecki | 2012 | ELISA | 3/25 | 12.0% | Including dogs with neurological signs | (36) |

*study included in the meta-analysis of seroprevalence

^a^ Note that the reference numbering is not identical to the main manuscript, as several references are only listed in this table.

^b^Germany, Austria, Czech Republic, Norway, Switzerland

**References**

1. Roelandt S, Suin V, Riocreux F, Lamoral S, Van der Heyden S, Van der Stede Y, et al. Autochthonous tick-borne encephalitis virus-seropositive cattle in Belgium: A risk-based targeted serological survey. *Vector Borne Zoonotic Dis* (2014) 14:640-7. doi: 10.1089/vbz.2014.1576.

2. Tuomi J, Brummer-Korvenkontio M. Antibodies against viruses of the tick-borne encephalitis group in cattle sera in Finland. *Ann Med Exp Biol Fenn* (1965) 43:149-54. PubMed ID: 5893666.

3. Šikutová S, Hornok S, Hubálek Z, Doležálková I, Juřicová Z, Rudolf I. Serological survey of domestic animals for tick-borne encephalitis and Bhanja viru*s*es in northeastern Hungary. *Vet Microbiol* (2009) 135:267-71. doi: 10.1016/j.vetmic.2008.09.082.

4. Juceviciene A, Zygutiene M, Leinikki P, Brummer-Korvenkontio H, Salminen M, Han X, et al. Tick-borne encephalitis virus infections in Lithuanian domestic animals and ticks. *Scand J Infect Dis* (2005) 37:742-6. doi: 10.1080/00365540510012134.

5. van der Poel WHM, van der Heide R, Bakker D, Looff MD, Jong JD, van Manen N, et al. Attempt to detect evidence for tick-borne encephalitis virus in ticks and mammalian wildlife in the Netherlands. *Vector Borne Zoonotic Dis* (2005) 5:58-64. doi: 10.1089/vbz.2005.5.58.

6. Traavik T. Serological investigations indicating the existence of tick-borne encephalitis virus foci along the Norwegian coast *Acta Path Microbiol Scand Section B* (1973) 81B:138-42. doi: 10.1111/j.1699-0463.1973.tb02197.x.

7. Paulsen KM, Stuen S, das Neves CG, Suhel F, Gurung D, Soleng A, et al. Tick-borne encephalitis virus in cows and unpasteurized cow milk from Norway. *Zoonoses Public Health* (2019) 66:216-22. doi: 10.1111/zph.12554.

8. Cisak E, Wójcik-Fatla A, Sroka J, Zając V, Bilska-Zając E, Chmurzyńska E, et al. Prevalence of tick-borne encephalitis virus antibodies in domestic and game animals from eastern Poland. *Bull Vet Inst Pulawy* (2012) 56:275-8. doi: 10.2478/v10213-012-0049-6.

9. Klaus C, Beer M, Saier R, Schau U, Moog U, Hoffmann B, et al. Goats and sheep as sentinels for tick-borne encephalitis (TBE) virus – Epidemiological studies in areas endemic and non-endemic for TBE virus in Germany. *Ticks Tick Borne Dis* (2012) 3:27-37. doi: 10.1016/j.ttbdis.2011.09.011.

10. Frimmel S, Löbermann M, Feldhusen F, Seelmann M, Stiasny K, Süss J, et al. Detection of tick-borne encephalitis virus antibodies in sera of sheep and goats in Mecklenburg-Western Pomerania (north-eastern Germany). *Ticks Tick Borne Dis* (2019) 10:901-4. doi: 10.1016/j.ttbdis.2019.04.012.

11. Hubálek Z, Mitterpák J, Prokopic J, Juricová Z, Kilík J. A serological survey for Bhanja and tick-borne encephalitis viruses in sheep of eastern Slovakia. *Folia Parasitol (Praha)* (1985) 32:279-83. PubMed ID: 3930351.

12. Grandi G, Aspán A, Pihl J, Gustafsson K, Engström F, Jinnerot T, et al. Detection of tick-borne pathogens in lambs undergoing prophylactic treatment against ticks on two Swedish farms. *Front Vet Sci* (2018) 5:6. doi: 10.3389/fvets.2018.00072.

13. Wallenhammar A, Lindqvist R, Asghar N, Gunaltay S, Fredlund H, Davidsson Å, et al. Revealing new tick-borne encephalitis virus foci by screening antibodies in sheep milk. *Parasit Vectors* (2020) 13:185. doi: 10.1186/s13071-020-04030-4.

14. Holzmann H, Aberle SW, Stiasny K, Werner P, Mischak A, Zainer B, et al. Tick-borne encephalitis from eating goat cheese in a mountain region of Austria. *Emerg Infect Dis* (2009) 15:1671-3. doi: 10.3201/eid1510.090743.

15. Klaus C, Ziegler U, Hoffmann D, Press F, Fast C, Beer M. Tick-borne encephalitis virus (TBEV) antibodies in animal sera – occurrence in goat flocks in Germany, longevity and ability to recall immunological information after more than six years. *BMC Vet Res* (2019) 15:399. doi: 10.1186/s12917-019-2157-5.

16. Klaus C, Ziegler U, Kalthoff D, Hoffmann B, Beer M. Tick-borne encephalitis virus (TBEV) – findings on cross reactivity and longevity of TBEV antibodies in animal sera. *BMC Vet Res* (2014) 10:78. doi: 10.1186/1746-6148-10-78.

17. Stefanoff P, Siennicka J, Kaba J, Nowicki M, Ferenczi E, Gut W. Identification of new endemic tick-borne encephalitis foci in Poland – a pilot seroprevalence study in selected regions. *Int J Med Microbiol* (2008) 298:102-7. doi: 10.1016/j.ijmm.2008.04.002.

18. Rieille N, Klaus C, Hoffmann D, Péter O, Voordouw MJ. Goats as sentinel hosts for the detection of tick-borne encephalitis risk areas in the Canton of Valais, Switzerland. *BMC Vet Res* (2017) 13:217. doi: 10.1186/s12917-017-1136-y.

19. Casati Pagani S, Frigerio Malossa S, Klaus C, Hoffmann D, Beretta O, Bomio-Pacciorini N, et al. First detection of TBE virus in ticks and sero-reactivity in goats in a non-endemic region in the southern part of Switzerland (Canton of Ticino). *Ticks Tick Borne Dis* (2019) 10:868-74. doi: 10.1016/j.ttbdis.2019.04.006.

20. Luckschander N. Frühsommermeningoenzephalitis (FSME) in einer österreichischen Pferdepopulation. *Tierarztl Prax Ausg G Großtiere* (1999) 27:235-8.

21. Rushton JO, Lecollinet S, Hubálek Z, Svobodová P, Lussy H, Nowotny N. Tick-borne encephalitis virus in horses, Austria, 2011. *Emerg Infect Dis* (2013) 19:635-7. doi: 10.3201/eid1904.121450.

22. Klaus C, Hörügel U, Hoffmann B, Beer M. Tick-borne encephalitis virus (TBEV) infection in horses: Clinical and laboratory findings and epidemiological investigations. *Vet Microbiol* (2013) 163:368-72. doi: 10.1016/j.vetmic.2012.12.041.

23. Janitza-Futterer D. Serologische Untersuchungen zur endemischen Situation der Infektion mit dem FSME-Virus in einer südbadischen Pferde-und Hundepopulation [Dissertation]. Munich: Ludwig-Maximilians-Universität München (2003).

24. Müller K, König M, Thiel HJ. Tick-borne encephalitis (TBE) with special emphasis on infection in horses. *Dtsch Tierarztl Wochenschr* (2006) 113:147-51. PubMed ID: 16716050.

25. Csank T, Drzewnioková P, Korytár Ľ, Major P, Gyuranecz M, Pistl J, et al. A serosurvey of flavivirus infection in horses and birds in Slovakia. *Vector Borne Zoonotic Dis* (2018) 18:206-13. doi: 10.1089/vbz.2017.2216.

26. Breu D, Guthardt J, Müller E. Seroprevalence of antibodies to tick-borne encephalitis virus in 433 dogs with neurological signs. *26th ECVIM-CA Congress*; Gothenburg, Sweden (2016).

27. Kirtz G. Frühsommer-Meningo-Encephalitis (FSME) in einer österreichischen Hundepopulation [Dissertation]. Vienna: Veterinärmedizinische Universität Wien (1999).

28. Roelandt S, Heyman P, De Filette M, Vene S, Van der Stede Y, Caij AB, et al. Tick-borne encephalitis virus seropositive dog detected in Belgium: Screening of the canine population as sentinels for public health. *Vector Borne Zoonotic Dis* (2011) 11:1371-6. doi: 10.1089/vbz.2011.0647.

29. Klimeš J, Literák I, Schánilec P, Juřicová Z, Trachta e Silva E. Prevalence of antibodies to tickborne encephalitis and West Nile flaviviruses and the clinical signs of tickborne encephalitis in dogs in the Czech Republic. *Vet Rec* (2001) 148:17. doi: 10.1136/vr.148.1.17.

30. Hekrlová A, Kubíček O, Lány P, Rosenbergová K, Schánilec P. Tick-borne encephalitis in dogs: application of "nested real-time RT-PCR" for intravital virus detection. *Berl Munch Tierarztl Wochenschr* (2015) 128:397-401. doi: 10.23767/0005-9366-128-397.

31. Lindhe KES, Meldgaard DS, Jensen PM, Houser GA, Berendt M. Prevalence of tick-borne encephalitis virus antibodies in dogs from Denmark. *Acta Vet Scand* (2009) 51:56. doi: 10.1186/1751-0147-51-56.

32. Levanov L, Vera CP, Vapalahti O. Prevalence estimation of tick-borne encephalitis virus (TBEV) antibodies in dogs from Finland using novel dog anti-TBEV IgG MAb-capture and IgG immunofluorescence assays based on recombinant TBEV subviral particles. *Ticks Tick Borne Dis* (2016) 7:979-82. doi: 10.1016/j.ttbdis.2016.05.002.

33. Balling A, Beer M, Gniel D, Pfeffer M. Prevalence of antibodies against tick-borne encephalitis virus in dogs from Saxony, Germany. *Berl Munch Tierarztl Wochenschr* (2015) 128:297-303. PubMed ID: 26281442.

34. Reiner B, Grasmück S, Steffen F, Djuric N, Schindler T, Müller W, et al. Prevalence of TBE antibodies in serum and CSF of dogs with inflammatory and non-inflammatory CNS disease. *Int J Med Microbiol* (2002) 291, Suppl. 33:234.

35. Csángó PA, Blakstad E, Kirtz GC, Pedersen JE, Czettel B. Tick-borne encephalitis in Southern Norway. *Emerging Infectious Disease journal* (2004) 10:533. doi: 10.3201/eid1003.020734.

36. Bajer A, Mierzejewska EJ, Rodo A, Bednarska M, Kowalec M, Welc-Falęciak R. The risk of vector-borne infections in sled dogs associated with existing and new endemic areas in Poland: Part 1: A population study on sled dogs during the racing season. *Vet Parasitol* (2014) 202:276-86. doi: 10.1016/j.vetpar.2013.12.033.
