# Supplementary material for: Zoonotic Tick-Borne Pathogens in Temperate and Cold Regions of Europe—A Review on the Prevalence in Domestic Animals
Source: Front Vet Sci. 2020 Dec 10;7:604910. doi: 10.3389/fvets.2020.604910 (PMC7758354; doi:10.3389/fvets.2020.604910)
Supplement: Supplementary file 2 [file Table_2.DOCX]

**Supplementary Table 2:** (Sero-)prevalence studies on *Borrelia burgdorferi* s.l. in domestic animals in temperate and cold regions of Europe. Abbreviations: ELISA: enzyme-linked immunosorbent assay; HAI: haemagglutination inhibition test; RT-PCR: reverse transcription polymerase chain reaction; RLB: reverse line blot; WB: Western blot.

| Country | Region | Year(s) of sampling | Method(s) | Positive/total | Prevalence | Comment(s) | Reference^a^ |
| --- | --- | --- | --- | --- | --- | --- | --- |
| Cattle | | | | | | | |
| France | Gironde | 1994 | IFAT | 96/364 | 26.4% |  | (1)* |
| Germany | Southern Germany | 1985 | IFAT | 22/66 | 33.3% |  | (2)* |
|  | Northern Germany | 1988-1989 | IFAT, ELISA | 63/427 (IFAT),  149/2451 (ELISA) | 14.8% (IFAT),  6.1% (ELISA) |  | (3)* |
|  | Northern Germany | 1988-1989 | IFAT | 172/1262 | 13.6% |  | (4)* |
|  | Northern Germany | 1988-1990 | IFAT | 202/554 | 36.5% | Farms with suspected/ history of babesiosis | (5)* |
|  | Berlin | 1988 | IFAT, ELISA | 13/53 (IFAT),  35/53 (ELISA) | 24.5% (IFAT),  66.0% (ELISA) | *B. burgdorferi* s.s. used as antigen | (6)* |
|  | Southern Germany | 1988 | IFAT, ELISA | 28/141 (IFAT),  61/141 (ELISA) | 19.8%, (IFAT),  43.3% (ELISA) | *B. burgdorferi* s.s. used as antigen | (6)* |
|  | Bavaria | 2002 | IFAT | 131/287 | 45.6% |  | (7)* |
| Poland | Nationwide | 2001-2003 | ELISA | 7/26 | 26.9% | *B. burgdorferi* s.s. + *B. afzelii* used as antigen; symptomatic^b^ cattle | (8) |
|  | Lublin | 1993-2007 | ELISA | 17/92 | 18.5% | *B. burgdorferi* s.s. + *B. garinii* + *B. afzelii* used as antigen | (9) |
| Slovakia | Different regions | 1979-2001 | ELISA | 227/973 | 25.2% | *B. garinii* used as antigen | (10) |
|  | Different regions | 1993-2007 | ELISA | 235/1043 | 22.5% | *B. burgdorferi* s.s. + *B. garinii* + *B. afzelii* used as antigen | (9) |
| Sweden | Northern Sweden |  | IFAT | 1/88 | 1.1% |  | (11)* |
|  | Southern Sweden |  | IFAT | 23/58 | 39.7% | Animals with elevated *Babesia* antibody titres | (11) |
|  | Southern Sweden |  | IFAT | 14/68 | 20.6% | Animals after the first grazing season | (11)* |
| Switzerland | NA | 2000 | ELISA  (*B. burgdorferi* s.s., *B. garinii*, *B. afzelii*) | 94/396  (*B. burgdorferi* s.s.),  64/396 (*B. garinii*),  57/396 (*B. afzelii*) | 23.7%  (*B. burgdorferi* s.s.),  16.2% (*B. garinii*),  14.4% (*B. afzelii*) |  | (12) |
| Sheep | | | | | | | |
| France | Gironde | 1994 | IFAT | 363/642 | 56.5% |  | (1)* |
| Norway | Nationwide | 1988-1989 | ELISA | 32/327 | 9.8% |  | (13)* |
| Sweden | Gotland | NA | IFAT | 11/13 | 84.6% | Lambs with arthritis | (11) |
|  | Jämtland | NA | IFAT | 0/10 | 0.0% |  | (11)* |
|  | Östergötland, Gotland | 2013 | IFAT | 2/43 | 4.7% | Farms with high lamb morbidity and mortality; *B. afzelii* used as antigen | (14) |
| Slovakia | Zemplínska Teplica | 1993-2007 | ELISA | 30/181 | 16.6% | *B. burgdorferi* s.s. + *B. garinii* + *B. afzelii* used as antigen | (9) |
| Goats | | | | | | | |
| Slovakia | Zemplínska Teplica | 1993-2007 | ELISA | 5/29 | 17.2% | *B. burgdorferi* s.s. + *B. garinii* + *B. afzelii* used as antigen | (9) |
| Horses | | | | | | | |
| Denmark | Nationwide | 2009 | Rapid ELISA  (C6 antigen) | 113/390 | 29.0% |  | (15)* |
| France | Centre-West | NA | Rapid ELISA  (C6 antigen) | 44/144 | 30.6% |  | (16)* |
|  | East | NA | Rapid ELISA  (C6 antigen) | 77/159 | 48.4% |  | (16)* |
|  | South-East | NA | Rapid ELISA  (C6 antigen) | 13/105 | 12.4% |  | (16)* |
| Germany | Berlin | 1988 | IFAT, ELISA | 0/224 (IFAT),  36/224 (ELISA) | 0.0% (IFAT),  16.1% (ELISA) | *B. burgdorferi s.s.* and German isolate 2/B45 used as antigen | (6)* |
| Poland | Nationwide | 2001-2003 | ELISA | 101/395 | 25.6% | *B. burgdorferi* s.s. and *B. afzelii* used as antigen | (8)* |
|  | Lublin | 1993-2007 | ELISA | 80/260 | 30.8% |  | (9) |
| Slovakia | Different regions | 1993-2007 | ELISA | 99/374 | 26.5% |  | (9) |
| Sweden | Nationwide | 1997-1998 | IFAT | 66/400 (group 1), 274/1618 (group 2) | 16.5% (group 1), 16.9% (group 2) | Group 1: healthy horses, group 2: non-healthy horses | (17)* |
| Dogs | | | | | | | |
| Austria | Nationwide | 2010-2011 | Rapid ELISA  (C6 antigen) | 12/254 | 4.7% | Symptomatic^b^ dogs | (18) |
| Czech Republic | Different regions | 2001-2002 | ELISA | 26/399 | 6.5% | Healthy military dogs; *B. afzelii* and *B. garinii* used as antigen | (19) |
|  | Nationwide | 2005-2007 | ELISA, PCR | 13/141 (ELISA, group 1),  17/155 (ELISA, group 2),  1/141 (PCR, group 1), 0/155 (PCR, group 2) | 9.2% (ELISA, group 1),  11.0% (ELISA, group 2),  0.7% (PCR, group 1),  0.0% (PCR, group 2) | Group 1: symptomatic^b^ dogs,  group 2: asymptomatic dogs | (20) |
| Denmark | Nationwide | 1986-1987 | IFAT | 33/205 | 16.1% |  | (21)* |
| Finland | Nationwide | 2010-2012 | Rapid ELISA  (C6 antigen) | 10/340 (group 1),  1/50 (group 2) | 2.9% (group 1),  2.0% (group 2) | Group 1: dogs presented at veterinary clinics, group 2: healthy hunting dogs | (22)* |
| France | Southern France | 1992 | ELISA + WB | 19/183 | 10.4% | Military dogs | (23)* |
|  | Nationwide | 2006 | Rapid ELISA  (C6 antigen) | 10/919 | 1.1% | Random diagnostic samples | (24)* |
| Germany | Southern Germany | 1985 | IFAT | 44/72 | 61.1% | Symptomatic^b^ dogs | (2) |
|  | Berlin | 1988 | IFAT, ELISA | 11/189 (IFAT),  19/189 (ELISA) | 5.8% (IFAT),  10.1% (ELISA) | *B. burgdorferi s.s.* and German isolate 2/B45 used as antigen | (6) |
|  | Bavaria | 1988-1990 | IFAT | 46/130 | 35.5% | Veterinary patients with various clinical signs | (25) |
|  | NA | 1993-1994 | ELISA + WB | 12/665 | 1.8% | *B. burgdorferi* s.s. used as antigen | (26)* |
|  | Nationwide | 2004-2007 | Rapid ELISA  (C6 antigen) | 232/3005 (group 1), 340/2876 (group 2) | 7.7% (group 1), 11.8% (group 2) | Group 1: random diagnostic samples, group 2: pre-selected for symptoms of borreliosis | (27)* |
|  | Munich | 2006-2008 | Rapid ELISA  (C6 antigen) | 22/448 | 4.9% | Includes dogs with various clinical signs | (28) |
|  | Southern Germany | NA | ELISA+WB | 74/171 (group 1),  10/57 (group 2) | 43.3% (group 1), 17.5% (group 2) | Group 1: Bernese Mountain dogs,  group 2: other breeds; WB to discriminate vaccinated from naturally infected dogs | (29)* |
| Hungary | Nationwide | 2011-2012 | Rapid ELISA  (C6 antigen) | 11/1305 | 0.8% | Randomly selected healthy dogs | (30)* |
| Latvia | Nationwide | 2009-2011 | Rapid ELISA  (C6 antigen) | 11/441 (group 1),  0/29 (group 2) | 2.5% (group 1),  0.0% (group 2) | Group 1: healthy dogs,  group 2: symptomatic^b^ dogs | (31)* |
| Lithuania | Different regions | 2016-2019 | Real-time PCR | 19/100 | 19.0% | Dogs with suspected babesiosis | (32) |
| Netherlands |  | 1989 | ELISA | 80/448 (group 1),  13/75 (group 2) | 17.9% (group 1), 17.3% (group 2) | Group 1: hunting dogs, group 2: pet dogs; *B. burgdorferi* s.s. used as antigen | (33)* |
|  |  | NA | IHA, ELISA | 66/501 (group 1, IHA), 84/501 (group1, ELISA),  90/676 (group 2, IHA), 128/676 (group 2, ELISA) | 13.2% (group 1, IHA), 16.8% (group 1, ELISA),  13.3% (group 2, IHA), 18.9% (group 2, ELISA) | Group 1: healthy dogs, group 2: symptomatic^b^ | (34)*^, †^ |
|  |  | NA | PCR + RLB (liver tissue) | 9/15 (group 1),  9/43 (group 2) | 60.0% (group 1), 20.9% (group 2) | Group 1: symptomatic^b^, group 2: asymptomatic for borreliosis;  *B. burgdorferi* s.s., *B. garinii*, *B. afzelii*, *B. valaisiana* detected | (35) |
| Norway | West Agder, East Agder | 1994-1995 | IFAT | 40/149 | 26.8% | Includes symptomatic^b^ dogs | (36) |
| Poland | Warsaw | 2003-2004 | PCR | 7/408 | 1.7% | Random diagnostic samples | (37) |
|  | Warsaw | 2006-2008 | PCR | 1/109 | 0.9% | Includes symptomatic^b^ dogs | (38) |
|  | Nationwide | 2011 | Rapid ELISA  (C6 antigen) | 115/3094 | 3.8% | Clinically healthy with tick history | (39)* |
|  | NA | NA | PCR | 6/15 | 40.0% | Symptomatic^b^ dogs | (40) |
|  | Northwestern Poland | NA | PCR + RFLP | 16/98 | 16.3% | Symptomatic^b^ dogs, *B. burgdorferi* s.s. identified in all samples | (41) |
|  | Szczecin | NA | ELISA, PCR | 37/92 (ELISA), 31/92 (PCR) | 40.2% (ELISA), 33.7% (PCR) | Symptomatic^b^ dogs, *B. burgdorferi* s.s. + *B. garinii* + *B. afzelii* used as antigen | (42) |
|  | Eastern Poland | 2011-2014 | Rapid ELISA  (C6 antigen), PCR | 44/400 (ELISA),  20/400 (PCR) | 11.0% (ELISA),  5.0% (PCR) | *B. afzelii* identified in all samples | (43)* |
| Sweden | Götaland | 1991-1994 | IFAT | 11/186 | 5.9% | Suspected scabies | (44)* |
|  | Svealand | 1991-1994 | IFAT | 12/306 | 3.9% | Suspected scabies | (44)* |
|  | Norrland | 1991-1994 | IFAT | 0/96 | 0.0% | Suspected scabies | (44)* |
|  | NA | 1993-2001 | IFAT | 21/243 | 8.6% | Dogs with neurological signs; *B. afzelii* used as antigen | (45) |
|  | NA | 2002-2005 | IFAT, PCR | 4/54 (IFAT),  0/54 (PCR) | 7.4% (IFAT),  0.0% (PCR) | Dogs with neurological signs | (46) |
|  | Nationwide | 2001 | PCR | 5/120 | 4.2% | Systemically ill dogs | (47) |
| Slovakia | Southern Slovakia | NA | Rapid ELISA  (C6 antigen) | 5/180 | 2.8% | Includes dogs with various clinical signs | (48) |
|  | Košice, Dubnica nad Váhom | 1993-2007 | ELISA | 283/846 | 33.5% |  | (9) |
|  | Eastern Slovakia | 2001-2002 | ELISA | 42/138 | 30.4% | Hunting dogs;  *B. burgdorferi* s.s., *B. garinii* + *B. afzelii* used as antigens | (49) |
| Switzerland | Nationwide | 2002-3003 | ELISA+WB | 92/160 (group 1),  9/61 (group 2) | 57.5% (group 1), 14.8% (group 2) | Group 1: Bernese Mountain dogs,  group 2: other breeds;  *B. burgdorferi* s.s. used as antigen | (50) |
|  | NA | NA | IFAT | 24/237 (group 1), 0/176 (group 2) | 10.1% (group 1), 0.0% (group 2) | Group 1: dogs with tick-bite history,  group 2: dogs without tick-bite history | (51) |
|  | NA | NA | ELISA | 21/98 (group 1),  4/55 (group 2),  15/236 (group 3) | 21.4% (group 1),  7.3% (group 2),  6.4% (group 3) | Group 1: symptomatic^b^,  group 2: healthy dogs, group 3: clinical signs not compatible with borreliosis | (52) |
| Cats | | | | | | | |
| Different European countries^c^ |  | 2009-2011 | Rapid ELISA  (C6 antigen) | 6/271 | 2.2% | Suspected borreliosis | (53) |
| Czech Republic | Brno | 2013-2015 | ELISA  (IgM + IgG) | 50/260 (group 1),  6/42 (group 2),  9/58 (group 3) | 19.2% (group 1), 14.3% (group 2), 15.8% (group 3) | Group 1: cats presented at veterinary clinics,  group 2: shelter cats,  group 3: street cats | (54)* |
| United Kingdom | Nationwide | 2001 | PCR | 2/120 | 1.6% | Systemically ill cats | (47) |

*study included in the meta-analysis of seroprevalence

^†^values determined by ELISA included in the meta-analysis

^a^ Note that the reference numbering is not identical to the main manuscript, as several references are only listed in this table.

^b^symptoms attributable to Lyme borreliosis

^c^Belgium, Denmark, Germany, Netherlands, Norway, Sweden, United Kingdom

**References**

1. Cabannes A, Hernandez JC, Lucchese F, Appriou M, Tribouley-Duret J. Epidemiology of Lyme disease in cattle and sheep, in Gironde (France). *Med Mal Infect* (1997) 27:878-83. doi: 10.1016/S0399-077X(97)80242-9.

2. Bark S. Zur Diagnose und Verbreitung der einheimischen Zecken-Borreliose beim Tier [Dissertation]. Munich, Germany: Ludwig-Maximilians-Universität München (1986).

3. Delfmann A. Untersuchung von Rinderseren auf Zeckenborreliose im Regierungsbezirk Weser-Ems mit Hilfe von IFAT und ELISA [Dissertation]. Hanover, Germany: University of Veterinary Medicine Hannover (1991).

4. Brand A. Vergleichende seroepidemiologische Untersuchung von Rindern auf Zeckenborreliose in der Südheide und im Weserbergland [Dissertation]. Hanover, Germany: University of Veterinary Medicine Hannover (1990).

5. Niepold J. Untersuchung von Rinderseren auf das simultane Vorkommen von Infektionen mit Borrelien (*Borrelia burgdorferi*) und Babesien (*Babesia divergens*). [Dissertation]. Hanover: University of Veterinary Medicine Hannover (1990).

6. Käsbohrer A, Schönberg A. Serologic studies of the occurrence of *Borrelia burgdorferi* in domestic animals in Berlin (West). *Berl Munch Tierarztl Wochenschr* (1990) 103:374-8. PubMed ID: 2268252.

7. Lengauer H, Just FT, Edelhofer R, Pfister K. Tick infestation and the prevalence of *Borrelia burgdorferi* and *Babesia divergens* in cattle in Bavaria. *Berl Munch Tierarztl Wochenschr* (2006) 119:335-41. PubMed ID: 17009719.

8. Štefančíková A, Adaszek Ł, Peťko B, Winiarczyk S, Dudiňák V. Serological evidence of *Borrelia burgdorferi* sensu lato in horses and cattle from Poland and diagnostic problems of Lyme borreliosis. *Ann Agric Environ Med* (2008) 15:37-43. PubMed ID.

9. Štefančíková A, Derdáková M, Škardová I, Szestáková E, Čisláková L, Kováčová D, et al. Some epidemiological and epizootiological aspects of Lyme borreliosis in Slovakia with the emphasis on the problems of serological diagnostics. *Biologia* (2008) 63:1135-42. doi: 10.2478/s11756-008-0177-x.

10. Štefančíková A, Štěpánová G, Derdáková M, Pet'ko B, Kysel'ová J, Cigánek J, et al. Serological evidence for *Borrelia burgdorferi* infection associated with clinical signs in dairy cattle in Slovakia. *Vet Res Commun* (2002) 26:601-11. doi: 10.1023/A:1020912618950.

11. Hovmark A, Åsbrink E, Schwan O, Hederstedt B, Christensson D. Antibodies to *Borrelia* spirochetes in sera from Swedish cattle and sheep. *Acta Vet Scand* (1986) 27:479-85. PubMed ID: 3604822.

12. Müller C. Seroprevalence of *Borrelia burgdorferi* sensu lato infection in cattle in an area of Switzerland with previously reported clinical cases [Dissertation]. Zurich: University of Zurich (2003).

13. Fridriksdóttir V, Nesse LL, Gudding R. Seroepidemiological studies of *Borrelia burgdorferi* infection in sheep in Norway. *J Clin Microbiol* (1992) 30:1271. doi: 10.1128/JCM.30.5.1271-1277.1992

14. Grandi G, Aspán A, Pihl J, Gustafsson K, Engström F, Jinnerot T, et al. Detection of tick-borne pathogens in lambs undergoing prophylactic treatment against ticks on two Swedish farms. *Front Vet Sci* (2018) 5:6. doi: 10.3389/fvets.2018.00072.

15. Hansen MGB, Christoffersen M, Thuesen LR, Petersen MR, Bojesen AM. Seroprevalence of *Borrelia burgdorferi* sensu lato and *Anaplasma phagocytophilum* in Danish horses. *Acta Vet Scand* (2010) 52:3. doi: 10.1186/1751-0147-52-3.

16. Maurizi L, Marié JL, Courtin C, Gorsane S, Chal D, Davoust B. Seroprevalence survey of equine anaplasmosis in France and in sub-Saharan Africa. *Clin Microbiol Infect* (2009) 15:68-9. doi: 10.1111/j.1469-0691.2008.02191.x.

17. Egenvall A, Franzén P, Gunnarsson A, Engvall EO, Vågsholm I, Wikström U-B, et al. Cross-sectional study of the seroprevalence to *Borrelia burgdorferi* sensu lato and granulocytic *Ehrlichia* spp. and demographic, clinical and tick-exposure factors in Swedish horses. *Prev Vet Med* (2001) 49:191-208. doi: 10.1016/S0167-5877(01)00187-8.

18. Pantchev N, Pluta S, Huisinga E, Nather S, Scheufelen M, Vrhovec MG, et al. Tick-borne diseases (borreliosis, anaplasmosis, babesiosis) in German and Austrian Dogs: Status quo and review of distribution, transmission, clinical findings, diagnostics and prophylaxis. *Parasitol Res* (2015) 114:19-54. doi: 10.1007/s00436-015-4513-0.

19. Pejchalová K, Žákovská A, Fučík K, Schánilec P. Serological confirmation of *Borrelia burgdorferi* infection in dogs in the Czech Republic. *Vet Res Commun* (2006) 30:231-8. doi: 10.1007/s11259-006-3214-7.

20. Kybicová K, Schánilec P, Hulínská D, Uherková L, Kurzová Z, Spejchalová S. Detection of *Anaplasma phagocytophilum* and *Borrelia burgdorferi* sensu lato in dogs in the Czech Republic. *Vector Borne Zoonotic Dis* (2009) 9:655-61. doi: 10.1089/vbz.2008.0127.

21. Hansen K, Dietz HH. Serosurvey for antibodies to *Borrelia burgdorferi* in Danish dogs. *APMIS* (1989) 97:281-5. doi: 10.1111/j.1699-0463.1989.tb00789.x.

22. Pérez Vera C, Kapiainen S, Junnikkala S, Aaltonen K, Spillmann T, Vapalahti O. Survey of selected tick-borne diseases in dogs in Finland. *Parasit Vectors* (2014) 7:285. doi: 10.1186/1756-3305-7-285.

23. Davoust B, Boni M. Borréliose de Lyme chez le chien : enquête séroépidémiologique dans le Sud-Est. *Med Mal Infect* (1998) 28:408-9. doi: 10.1016/S0399-077X(98)70242-2.

24. Pantchev N, Schaper R, Limousin S, Norden N, Weise M, Lorentzen L. Occurrence of *Dirofilaria immitis* and tick-borne infections caused by *Anaplasma phagocytophilum, Borrelia burgdorferi* sensu lato and *Ehrlichia canis* in domestic dogs in France: Results of a countrywide serologic survey. *Parasitol Res* (2009) 105:101-14. doi: 10.1007/s00436-009-1501-2.

25. Weber A, Heim U, Schäfer R. Incidence of antibodies to *Borrelia burgdorferi* in dogs in small animal practice in North Bavaria. *Berl Munch Tierarztl Wochenschr* (1991) 104:384-6. PubMed ID: 1772393.

26. Wittenbrink MM, Failing K, Krauss H. Enzyme-linked immunosorbent assay and immunoblot analysis for detection of antibodies to *Borrelia burgdorferi* in dogs: The impact of serum absorption with homologous and heterologous bacteriae. *Vet Microbiol* (1996) 48:257-68. doi: 10.1016/0378-1135(95)00165-4.

27. Krupka I, Pantchev N, Lorentzen L, Weise M, Straubinger RK. Durch Zecken übertragbare bakterielle Infektionen bei Hunden: Seroprävalenzen von *Anaplasma phagocytophilum*, *Borrelia burgdorferi* sensu lato und *Ehrlichia canis* in Deutschland. *Praktischer Tierarzt* (2007) 88:776-88.

28. Barth C, Straubinger RK, Sauter-Louis C, Hartmann K. Prevalence of antibodies against *Borrelia burgdorferi* sensu lato and *Anaplasma phagocytophilum* and their clinical relevance in dogs in Munich, Germany. *Berl Munch Tierarztl Wochenschr* (2012) 125:337-44. PubMed ID: 22919928.

29. Preyß-Jägeler C, Müller E, Straubinger RK, Hartmann K. Prävalenz von Antikörpern gegen *Borrelia burgdorferi*, *Anaplasma phagocytophilum* und bestimmte *Leptospira-interrogans*-Serovare bei Berner Sennenhunden. *Tierarztl Prax Ausg K Kleintiere Heimtiere* (2016) 44:77-85. doi: 10.15654/TPK-140962.

30. Farkas R, Gyurkovszky M, Lukács Z, Aladics B, Solymosi N. Seroprevalence of some vector-borne infections of dogs in Hungary. *Vector Borne Zoonotic Dis* (2014) 14:256-60. doi: 10.1089/vbz.2013.1469.

31. Berzina I, Capligina V, Bormane A, Pavulina A, Baumanis V, Ranka R, et al. Association between *Anaplasma phagocytophilum* seroprevalence in dogs and distribution of *Ixodes ricinus* and *Ixodes persulcatus* ticks in Latvia. *Ticks Tick Borne Dis* (2013) 4:83-8. doi: 10.1016/j.ttbdis.2012.08.003.

32. Radzijevskaja J, Tamoliūnaitė D, Sabūnas V, Aleksandravičienė A, Paulauskas A. Prevalence and co-infection of mosquito-and tick-borne pathogens in domestic dogs suspected for canine babesiosis in Lithuania. *Biologija* (2020) 66:94-102. doi: 10.6001/biologija.v66i2.4256

33. Goossens HAT, van den Bogaard AE, Nohlmans MKE. Dogs as sentinels for human Lyme Borreliosis in the Netherlands. *J Clin Microbiol* (2001) 39:844. doi: 10.1128/JCM.39.3.844-848.2001.

34. Goossens HA, Maes JH, van den Bogaard AE. The prevalence of antibodies against *B. burgdorferi*, an indicator for Lyme borreliosis in dogs? A comparison of serological tests. *Tijdschr Diergeneeskd* (2003) 128:650-7. PubMed ID: 14650723.

35. Hovius KE, Stark LA, Bleumink-Pluym NM, van de Pol I, Verbeek-de Kruif N, Rijpkema SG, et al. Presence and distribution of *Borrelia burgdorferi* sensu lato species in internal organs and skin of naturally infected symptomatic and asymptomatic dogs, as detected by polymerase chain reaction. *Vet Q* (1999) 21:54-8. doi: 10.1080/01652176.1999.9694992.

36. Csángó P, Stamberg P, Pedersen J. Antibodies to *Borrelia burgdorferi*. Antibodies in dogs in Agder counties of southern Norway. *Canine Pract* (1996) 21:27-8.

37. Zygner W, Górski P, Wędrychowicz H. Detection of the DNA of *Borrelia afzelii*, *Anaplasma phagocytophilum* and *Babesia canis* in blood samples from dogs in Warsaw. *Vet Rec* (2009) 164:465. doi: 10.1136/vr.164.15.465.

38. Welc-Falęciak R, Rodo A, Siński E, Bajer A. *Babesia cani*s and other tick-borne infections in dogs in central Poland. *Vet Parasitol* (2009) 166:191-8. doi: 10.1016/j.vetpar.2009.09.038.

39. Krämer F, Schaper R, Schunack B, Połozowski A, Piekarska J, Szwedko A, et al. Serological detection of *Anaplasma phagocytophilum, Borrelia burgdorferi* sensu lato and *Ehrlichia canis* antibodies and *Dirofilaria immitis* antigen in a countrywide survey in dogs in Poland. *Parasitol Res* (2014) 113:3229-39. doi: 10.1007/s00436-014-3985-7.

40. Skotarczak B, Wodecka B. Molecular evidence of the presence of *Borrelia burgdorferi* sensu lato in blood samples taken from dogs in Poland. *Ann Agric Environ Med* (2003) 10:113-5. PubMed ID: 12852742.

41. Skotarczak B, Wodecka B. Identification of *Borrelia burgdorferi* genospecies inducing Lyme disease in dogs from western Poland. *Acta Vet Hung* (2005) 53:13-21. doi: 10.1556/avet.53.2005.1.2.

42. Skotarczak B, Wodecka B, Rymaszewska A, Sawczuk M, Maciejewska A, Adamska M, et al. Prevalence of DNA and antibodies to *Borrelia burgdorferi* sensu lato in dogs suspected of borreliosis. *Ann Agric Environ Med* (2005) 12:199-205. PubMed ID: 16457474.

43. Dzięgiel B, Adaszek Ł, Carbonero A, Łyp P, Winiarczyk M, Dębiak P, et al. Detection of canine vector-borne diseases in eastern Poland by ELISA and PCR. *Parasitol Res* (2016) 115:1039-44. doi: 10.1007/s00436-015-4832-1.

44. Egenvall A, Bonnett BN, Gunnarsson A, Hedhammar A, Shoukri M, Bornstein S, et al. Sero-prevalence of granulocytic *Ehrlichia* spp. and *Borrelia burgdorferi* sensu lato in Swedish dogs 1991-94. *Scand J Infect Dis* (2000) 32:19-25. doi: 10.1080/00365540050164164.

45. Jäderlund KH, Egenvall A, Bergström K, Hedhammar Å. Seroprevalence of *Borrelia burgdorferi* sensu lato and *Anaplasma phagocytophilum* in dogs with neurological signs. *Vet Rec* (2007) 160:825. doi: 10.1136/vr.160.24.825.

46. Jäderlund KH, Bergström K, Egenvall A, Hedhammar Å. Cerebrospinal fluid PCR and antibody concentrations against *Anaplasma phagocytophilum* and *Borrelia burgdorferi* sensu lato in dogs with neurological signs. *J Vet Intern Med* (2009) 23:669-72. doi: 10.1111/j.1939-1676.2009.0313.x.

47. Shaw SE, Binns SH, Birtles RJ, Day MJ, Smithson RC, Kenny MJ. Molecular evidence of tick-transmitted infections in dogs and cats in the United Kingdom. *Vet Rec* (2005) 157:645. doi: 10.1136/vr.157.21.645.

48. Čabanová V, Pantchev N, Hurníková Z, Miterpáková M. Recent study on canine vector-borne zoonoses in southern Slovakia - serologic survey. *Acta Parasitol* (2015) 60:749-58. doi: 10.1515/ap-2015-0107.

49. Bhide MR, Curlik J, Travnicek M, Lazar P. Protein A/G dependent ELISA a promising diagnostic tool in Lyme disease seroprevalence in game animals and hunting dogs. *Comp Immunol Microbiol Infect Dis* (2004) 27:191-9. doi: 10.1016/j.cimid.2003.10.001.

50. Gerber B, Eichenberger S, Wittenbrink MM, Reusch CE. Increased prevalence of *Borrelia burgdorferi* infections in Bernese Mountain Dogs: a possible breed predisposition. *BMC Vet Res* (2007) 3:15. doi: 10.1186/1746-6148-3-15.

51. Pfister K, Bigler B, Neswadba J, Gern L, Aeschlimann A. *Borrelia burgdorferi* infections of dogs in Switzerland. *Zbl Bakt* (1989) Suppl. 18:26-31.

52. Speck S, Reiner B, Streich WJ, Reusch C, Wittenbrink MM. Canine borreliosis: A laboratory diagnostic trial. *Vet Microbiol* (2007) 120:132-41. doi: 10.1016/j.vetmic.2006.10.017.

53. Pantchev N, Vrhovec MG, Pluta S, Straubinger RK. Seropositivity of *Borrelia burgdorferi* in a cohort of symptomatic cats from Europe based on a C6-peptide assay with discussion of implications in disease aetiology. *Berl Munch Tierarztl Wochenschr* (2016) 129:333-9. doi: 10.2376/0005-9366-15088.

54. Žákovská A, Schánilec P, Treml F, Dušková M, Agudelo RCF. Seroprevalence of Antibodies against *Borrelia burgdorferi* s. l. and *Leptospira interrogans* s. l. in cats in district of Brno and its environs, the Czech Republic. *Ann Agric Environ Med* (2020). doi: 10.26444/aaem/122804.
