# Supplementary material for: Zoonotic Tick-Borne Pathogens in Temperate and Cold Regions of Europe—A Review on the Prevalence in Domestic Animals
Source: Front Vet Sci. 2020 Dec 10;7:604910. doi: 10.3389/fvets.2020.604910 (PMC7758354; doi:10.3389/fvets.2020.604910)
Supplement: Supplementary file 3 [file Table_3.docx]

**Supplementary Table 3:** (Sero-)prevalence studies on *A. phagocytophilum* in domestic animals in temperate and cold regions of Europe. Abbreviations: ELISA: enzyme-linked immunosorbent assay; IFAT: immunofluorescence antibody test; HRM PCR: high-resolution melt PCR; RLB: reverse line blot.

| Country | Region | Year(s) of sampling | Method(s) | Positive/total | Prevalence | Comment(s) | Reference^a^ |
| --- | --- | --- | --- | --- | --- | --- | --- |
| Cattle | | | | | | | |
| Belgium | NA | 2010 | IFAT | 20/65 (spring),  50/65 (summer),  37/65 (autumn) | 30.8% (spring),  77.0% (summer),  56.9% (autumn) | Farms with a known history of anaplasmosis/babesiosis | (1) |
| Czech Republic | Bohemia | 2002-2003 | IFAT/  Real-time PCR | 3/55 (IFAT),  3/55 (PCR) | 5.5% (IFAT),  5.5% (PCR) |  | (2)* |
| Norway | Northern Norway | 2004 | IFAT | 14/14 (group 1),  4/18 (group 2) | 100.0% (group 1), 22.2% (group 2) | Group 1: farm with anaplasmosis outbreak,  group 2: healthy animals | (3)* |
| Sweden | Southern Sweden | 1994-1995 | PCR | 6/7 | 85.7% | Symptomatic^b^ animals | (4) |
|  | Southern Sweden | NA | Real-time PCR | 17/71 | 23.9% | Includes 39 cattle with symptoms of babesiosis | (5) |
| Switzerland |  | 1996-1997 | IFAT | 11-44/70 | 16.0-63.0% | Animals sampled monthly for one year | (6) |
| Sheep | | | | | | | |
| Czech Republic | Moravia | 1999 | IFAT | 41/41 (group 1),  8/17 (group 2) | 100% (group 1), 47.1% (group 2) | Group 1: herd responsible for an alimentary TBEV outbreak, group 2: other herds | (7)* |
| Czech Republic/  Slovakia | Different regions | 2006-2010 | PCR | 9/311 | 2.9% |  | (8) |
| Denmark | Jutland | 2000 | PCR | 2/25 | 8.0% |  | (9) |
|  | Zealand | 2006 | PCR | 3/12 | 25.0% | Flock suspected of anaplasmosis outbreak | (9) |
| Germany | Nationwide | 2006 | PCR | 11/255 | 4.3% | Samples for Q-fever surveillance | (10) |
| Norway | Nationwide | 1996-1997 | IFAT | 270/749 | 36.0% |  | (11)* |
|  | Mid-Norway | 2007-2008 | IFAT | 664/1208 | 55.0% |  | (12)* |
| Sweden | Östergötland, Gotland | 2013 | IFAT, real-time PCR | 26/43 (IFAT),  18/43 (PCR) | 60.5% (IFAT),  41.9% (PCR) | Farms with high lamb morbidity and mortality | (13) |
| Goats | | | | | | | |
| Switzerland | Central Switzerland | 2008 | real-time PCR | 4/72 | 5.6% |  | (14) |
| United Kingdom | Northern Ireland | NA | PCR | 4/5 | 80.0% | Feral goats | (15) |
| Horses | | | | | | | |
| Czech Republic | Bohemia | 2002-2003 | IFAT/  Real-time PCR | 2/40 (IFAT),  2/40 (PCR) | 5.0% (IFAT),  5.0% (PCR) |  | (2)* |
|  | Different regions | 2005-2007 | IFAT | 67/92 | 72.8% |  | (16)* |
| Denmark | Nationwide | 2009 | Rapid ELISA^c^ | 87/390 | 22.3% |  | (17)* |
| France | Centre-West | NA | Rapid ELISA^c^ | 23/144 | 16.0% |  | (18)* |
|  | East | NA | Rapid ELISA^c^ | 32/159 | 20.1% |  | (18)* |
|  | South-East | NA | Rapid ELISA^c^ | 0/105 | 0.0% |  | (18)* |
|  | Southern France | 2001-2002 | ELISA | 48/424 | 11.3% |  | (19)* |
| Germany | NA | NA | PCR | 1/6 | 16.7% | Thrombocytopenic horses | (20) |
| Netherlands |  | 2002-2005 | PCR + RLB | 6/61 | 9.8% | Horses with fever of unknown origin | (21) |
| Poland | Northern Poland | 2013 | PCR | 2/76 | 2.6% |  | (22) |
|  | NA | NA | PCR | 1/10 | 10.0% | Thrombocytopenic horses | (20) |
| Slovakia | Different regions | 2013 | PCR | 1/39 | 2.6% |  | (22) |
| Sweden | Southern Sweden | 1994-1995 | PCR | 18/26 | 69.2% | Symptomatic^b^ horses | (4) |
|  | Nationwide | 1997-1998 | IFAT | 62/400 (group 1), 274/1618 (group 2) | 15.5% (group 1),  16.9% (group 2) | Group 1: healthy horses,  group 2: non-healthy horses | (23)* |
| Switzerland | Nationwide | NA | IFAT | 66/1645 | 4.0% |  | (24)* |
| Ukraine | Different regions | 2013 | PCR | 0/100 | 0.0% |  | (22) |
|  | NA | NA | PCR | 6/36 | 16.7% | Thrombocytopenic horses | (20) |
| Dogs | | | | | | | |
| Austria | Nationwide | 2001-2006 | IFAT | 830/1470 | 56.5% |  | (25)* |
|  | Nationwide | 2010-2011 | Real-time PCR | 13/80 | 16.5% | Symptomatic^b^ dogs | (26) |
| Czech Republic | Nationwide | 2005-2007 | IFAT/PCR | 39/141 (IFAT, group 1),  38/155 (IFAT, group 2), 9/141 (PCR, group 1), 1/155 (PCR, group 2) | 27.5% (IFAT, group 1),  24.7% (IFAT, group 2),  6.4% (PCR, group 1), 0.6% (PCR, group 2) | Group 1: symptomatic^b^ dogs,  group 2: asymptomatic dogs | (27)* |
|  | NA | 2009-2012 | PCR | 2/19 | 10.5% | Dogs with fatal immunhaemolytic anaemia | (28) |
| Finland | Nationwide | 2010-2012 | Rapid ELISA^c^ | 18/340 (group 1),  2/50 (group 2) | 5.3% (group 1),  4.0% (group 2) | Group 1: dogs presented at veterinary clinics, group 2: healthy hunting dogs | (29)* |
| France | Nationwide | 2006 | Rapid ELISA^c^ | 25/919 | 2.7% |  | (30)* |
| Germany | Nationwide | NA | IFAT/PCR | 22/49 (group 1, IFAT), 26/62 (group 2, IFAT), 6/49 (group 1, PCR), 1/62 (group 2, PCR) | 44.9% (group 1, IFAT), 41.9% (group 2, IFAT), 12.2%, (group 1, PCR), 1.6% (group 2, PCR) | Group 1: symptomatic^b^ dogs, group 2: no signs of anaplasmosis | (31)* |
|  | Nationwide | 2005-2006 | IFAT/PCR | 121/258 (group 1, IFAT), 105/264 (group 2, IFAT), 20/258 (group 1, PCR), 10/264 (group 2, PCR) | 46.9% (group 1, IFAT), 39.8% (group 2, IFAT), 7.8% (group 1, PCR), 3.8% (group 2, PCR) | Group 1: symptomatic^b^ dogs, group 2: clinically healthy | (32)* |
|  | Nationwide | 2001-2005 | IFAT | 563/1124 | 50.1% | Anamnesis suggestive of anaplasmosis | (33) |
|  | Nationwide | 2004-2007 | Rapid ELISA^c^ | 660/3005 (group 1),  564/2678 (group 2) | 22.0% (group 1),  21.1% (group 2) | Group 1: random diagnostic samples,  group 2: suspected borreliosis | (34)* |
|  | NA | 2005-2007 | Real-time PCR | 22/258 | 8.5% | Symptomatic^b^ dogs | (35) |
|  | Berlin/Brandenburg | 2006-2012 | Real-time PCR | 21/517 | 4.1% |  | (36) |
|  | Berlin/Brandenburg | 2006-2012 | Real-time PCR | 72/974 | 7.4% | Symptomatic^b^ dogs | (37) |
|  | Brandenburg | 2013-2014 | HRM PCR | 15/1023 | 1.5% |  | (38) |
|  | Munich | 2006-2008 | Rapid ELISA^c^ | 78/448 | 19.4% | Includes dogs with various clinical signs | (39) |
|  | Southern Germany | NA | IFAT | 86/171 (group 1),  14/57 (group 2) | 50.3% (group 1),  24.6% (group 2) | Group 1: Bernese Mountain dogs, group 2: other breeds | (40)* |
| Germany/ Switzerland | North of the Alps | 2006 | IFAT | 47/245 (group 1), 87/271 (group 2) | 19.2% (group 1),  32.1% (group 2) | Group 1: healthy dogs,  group 2: suspected borreliosis | (41)* |
| Hungary | Nationwide | 2011-2012 | Rapid ELISA^c^ | 102/1305 | 7.9% |  | (42)* |
|  | Southwestern Hungary | 2017 | PCR | 1/90 | 1.1% | Randomly selected veterinary patients | (43) |
| Latvia | Nationwide | 2009-2011 | Rapid ELISA^c^ | 44/400 (group 1),  5/41 (group 2),  5/29 (group 3) | 11.0% (group 1),  12.2% (group 2),  17.2% (group 3) | group 1: healthy dogs,  group 2: healthy hunting dogs, group 3: symptomatic^b^ dogs | (44)* |
| Lithuania | Different regions | 2016-2019 | Real-time PCR | 35/100 | 35.0% | Suspected babesiosis | (45) |
| Poland | North-Western Poland | NA | PCR | 4/100 (group 1),  13/92 (group 2),  0/50 (group 3) | 4.0% (group 1),  14.1% (group 2),  0.0% (group 3) | Group 1: healthy shelter dogs, group 2: suspected borreliosis, group 3: diagnosed babesiosis | (46) |
|  | Warsaw | 2003-2004 | PCR | 2/408 | 0.5% |  | (47) |
|  | Warsaw | 2006-2008 | PCR | 1/109 | 0.9% | Includes symptomatic^b^ dogs | (48) |
|  | Nationwide | 2009-2010 | PCR | 0/126 | 0.0% |  | (49) |
|  | Nationwide | 2011 | Rapid ELISA^c^ | 381/3094 | 12.3% | Clinically healthy with tick history | (50)* |
|  | Eastern Poland | 2011-2014 | Rapid ELISA^c^/  PCR | 32/400 (ELISA),  11/400 (PCR) | 8.0% (ELISA),  2.8% (PCR) |  | (51)* |
| Slovakia | NA | NA | PCR | 12/366 | 3.3% | Microfilaremic dogs | (52) |
|  | Southern Slovakia | NA | Rapid ELISA^c^ | 21/180 | 11.7% | Includes dogs with various clinical signs | (53) |
| Sweden | NA | 1990-1992 | IFAT | 84/640 | 13.1% | Symptomatic^b^ | (54) |
|  | Götaland | 1991-1994 | IFAT | 41/186 | 22.0% | Suspected scabies | (55)* |
|  | Svealand | 1991-1994 | IFAT | 61/306 | 19.9% | Suspected scabies | (55)* |
|  | Norrland | 1991-1994 | IFAT | 2/96 | 2.1% | Suspected scabies | (55)* |
|  | Southern Sweden | 1994-1995 | PCR | 10/15 | 66.7% | Symptomatic^b^ dogs | (4) |
|  | NA | 1993-2001 | IFAT | 51/246 | 20.7% | Dogs with neurological signs | (56) |
|  | NA | 2002-2005 | IFAT, PCR | 8/54 (IFAT),  0/54 (PCR) | 14.8% (IFAT),  0.0% (PCR) | Dogs with neurological signs | (57) |
| Switzerland | North of the Alps | 1991-1998 | IFAT | 17/235 | 7.2% |  | (58)* |
|  | Nationwide | 2005-2006 | Real-time PCR | 0/889 | 0.0% |  | (59) |
| United Kingdom | Nationwide | 2001 | PCR | 1/120 | 0.8% | Systemically ill dogs | (60) |
| Cats | | | | | | | |
| Ireland | Dublin | 2008 | PCR | 0/121 | 0.0% |  | (61) |
| Germany | Southern Germany | NA | PCR | 2/479 | 0.4% | Presented to veterinary clinics for various reasons | (62) |
|  | NA | 2006-2008 | IFAT | 45/238 (group 1),  2/10 (group 2),  6/58 (group 3) | 18.9% (group 1),  20.0% (group 2),  10.3% (group 3) | Group 1: random diagnostic samples,  group 2: symptomatic^b^ cats, group 3: healthy shelter cats | (63)* |
|  | Berlin | 2006-2008 | HRM PCR | 6/141 | 4.3% | Spleen samples collected during necropsy from shelter cats, no information on cause of death | (64) |
|  | Berlin/Brandenburg | 2007-2008 | IFAT/  real-time PCR | 21/216 (IFAT, group 1),  3/49 (IFAT, group 2), 1/216 (PCR, group 1), 0/49 (PCR, group 2) | 9.7% (IFAT, group 1), 6.1% (IFAT, group 2), 0.5% (PCR, group 1), 0.0% (PCR, group 2) | Group 1: cats with different clinical symptoms,  group 2: healthy cats | (65)* |
| United Kingdom | Nationwide | 2001 | Real-time PCR | 1/60 | 0.8% | Systemically ill cats | (60) |

*study included in the meta-analysis of seroprevalence

^a^ Note that the reference numbering is not identical to the main manuscript, as several references are only listed in this table.

^b^symptoms attributable to *A. phagocytophilum* infection

^c^no differentiation between *A. phagocytophilum* and *A. platys* antibodies

**References**

1. Lempereur L, Lebrun M, Cuvelier P, Sépult G, Caron Y, Saegerman C, et al. Longitudinal field study on bovine *Babesia* spp. and *Anaplasma phagocytophilum* infections during a grazing season in Belgium. *Parasitol Res* (2012) 110:1525-30. doi: 10.1007/s00436-011-2657-0.

2. Hulínská D, Langrová K, Pejčoch M, Pavlásek I. Detection of *Anaplasma phagocytophilum* in animals by real-time polymerase chain reaction. *APMIS* (2004) 112:239-47. doi: 10.1111/j.1600-0463.2004.apm11204-0503.x.

3. Stuen S, Oppegaard AS, Bergström K, Moum T. *Anaplasma phagocytophilum* infection in north Norway. The first laboratory confirmed case. *Acta Vet Scand* (2005) 46:167. doi: 10.1186/1751-0147-46-167.

4. Engvall EO, Pettersson B, Persson M, Artursson K, Johansson KE. A 16S rRNA-based PCR assay for detection and identification of granulocytic *Ehrlichia* species in dogs, horses, and cattle. *J Clin Microbiol* (1996) 34:2170-4. doi: 10.1128/JCM.34.9.2170-2174.1996

5. Andersson MO, Víchová B, Tolf C, Krzyzanowska S, Waldenström J, Karlsson ME. Co-infection with *Babesia divergens* and *Anaplasma phagocytophilum* in cattle (*Bos taurus*), Sweden. *Ticks Tick Borne Dis* (2017) 8:933-5. doi: 10.1016/j.ttbdis.2017.08.005.

6. Pusterla N, Pusterla JB, Braun U, Lutz H. Serological, hematologic and PCR studies of cattle in an area of Switzerland in which tick-borne fever (caused by *Ehrlichia phagocytophila*) is endemic. *Clin Diagn Lab Immunol* (1998) 5:325-7. doi: 10.1128/cdli.5.3.325-327.1998.

7. Zeman P, Januska J, Orolinova M, Stuen S, Struhar V, Jebavy L. High seroprevalence of granulocytic ehrlichiosis distinguishes sheep that were the source of an alimentary epidemic of tick-borne encephalitis. *Wien Klin Wochenschr* (2004) 116:614-6. doi: 10.1007/s00508-004-0191-0.

8. Derdáková M, Štefančíková A, Špitalská E, Tarageľová V, Košťálová T, Hrkľová G, et al. Emergence and genetic variability of *Anaplasma* species in small ruminants and ticks from central Europe. *Vet Microbiol* (2011) 153:293-8. doi: 10.1016/j.vetmic.2011.05.044.

9. Kiilerich AM, Christensen H, Thamsborg SM. *Anaplasma phagocytophilum* in Danish sheep: confirmation by DNA sequencing. *Acta Vet Scand* (2009) 51:55. doi: 10.1186/1751-0147-51-55.

10. Scharf W, Schauer S, Freyburger F, Petrovec M, Schaarschmidt-Kiener D, Liebisch G, et al. Distinct host species correlate with *Anaplasma phagocytophilum* ankA gene clusters. *J Clin Microbiol* (2011) 49:790-6. doi: 10.1128/jcm.02051-10.

11. Stuen S, Bergström K. Serological investigation of granulocytic *Ehrlichia* infection in sheep in Norway. *Acta Vet Scand* (2001) 42:331-8. doi: 10.1186/1751-0147-42-331.

12. Grøva L, Olesen I, Steinshamn H, Stuen S. Prevalence of *Anaplasma phagocytophilum* infection and effect on lamb growth. *Acta Vet Scand* (2011) 53:30. doi: 10.1186/1751-0147-53-30.

13. Grandi G, Aspán A, Pihl J, Gustafsson K, Engström F, Jinnerot T, et al. Detection of tick-borne pathogens in lambs undergoing prophylactic treatment against ticks on two Swedish farms. *Front Vet Sci* (2018) 5:6. doi: 10.3389/fvets.2018.00072.

14. Silaghi C, Scheuerle MC, Friche Passos LM, Thiel C, Pfister K. PCR detection of *Anaplasma phagocytophilum* in goat flocks in an area endemic for tick-borne fever in Switzerland. *Parasite* (2011) 18:57-62. doi: 10.1051/parasite/2011181057.

15. Harrison A, Brown KJ, Ian Montgomery W. *Anaplasma phagocytophilum* in feral goats in Northern Ireland. *Vet Rec* (2012) 170:602-3. doi: 10.1136/vr.e3938.

16. Praskova I, Bezdekova B, Zeman P, Jahn P. Seroprevalence of *Anaplasma phagocytophilum* in horses in the Czech Republic. *Ticks Tick Borne Dis* (2011) 2:111-5. doi: 10.1016/j.ttbdis.2011.01.002.

17. Hansen MGB, Christoffersen M, Thuesen LR, Petersen MR, Bojesen AM. Seroprevalence of *Borrelia burgdorferi* sensu lato and *Anaplasma phagocytophilum* in Danish horses. *Acta Vet Scand* (2010) 52:3. doi: 10.1186/1751-0147-52-3.

18. Maurizi L, Marié JL, Courtin C, Gorsane S, Chal D, Davoust B. Seroprevalence survey of equine anaplasmosis in France and in sub-Saharan Africa. *Clin Microbiol Infect* (2009) 15:68-9. doi: 10.1111/j.1469-0691.2008.02191.x.

19. Leblond A, Pradier S, Pitel PH, Fortier G, Boireau P, Chadoeuf J, et al. Enquête epidémiologique sur l'anaplasmose equine (*Anaplasma phagocytophilum*) dans le Sud de la France. *Rev Sci Tech* (2005) 24:899-908. PubMed ID: 16642760.

20. Dzięgiel B, Adaszek L, Winiarczyk M, García-Bocanegra I, Carbonero A, Dębiak P, et al. Comparative analysis of 16S RNA nucleotide sequences of *Anaplasma phagocytophilum* detected in the blood of horses from various parts of Europe. *J Med Microbiol* (2013) 62:1891-6. doi: 10.1099/jmm.0.058636-0.

21. Butler CM, Nijhof AM, Jongejan F, van der Kolk JH. *Anaplasma phagocytophilum* infection in horses in the Netherlands *Vet Rec* (2008) 162:216-8. doi: 10.1136/vr.162.7.216.

22. Slivinska K, Víchová B, Werszko J, Szewczyk T, Wróblewski Z, Peťko B, et al. Molecular surveillance of *Theileria equi* and *Anaplasma phagocytophilum* infections in horses from Ukraine, Poland and Slovakia. *Vet Parasitol* (2016) 215:35-7. doi: 10.1016/j.vetpar.2015.10.025.

23. Egenvall A, Franzén P, Gunnarsson A, Engvall EO, Vågsholm I, Wikström U-B, et al. Cross-sectional study of the seroprevalence to *Borrelia burgdorferi* sensu lato and granulocytic *Ehrlichia* spp. and demographic, clinical and tick-exposure factors in Swedish horses. *Prev Vet Med* (2001) 49:191-208. doi: 10.1016/S0167-5877(01)00187-8.

24. Bretscher R. Serologische Untersuchungen zur Häufigkeit und Verbreitung der equinen Ehrlichiose in der Schweiz. *Schweiz Arch Tierheilkd* (1991) 133:185.

25. Kirtz G, Czettel B, Thum D, Leidinger E. *Anaplasma phagocytophilum* in einer österreischichen Hundepopulation: eine Prävalenz-Studie (2001–2006). *Kleintierpraxis* (2007) 7:562-8.

26. Pantchev N, Pluta S, Huisinga E, Nather S, Scheufelen M, Vrhovec MG, et al. Tick-borne diseases (borreliosis, anaplasmosis, babesiosis) in German and Austrian Dogs: Status quo and review of distribution, transmission, clinical findings, diagnostics and prophylaxis. *Parasitol Res* (2015) 114:19-54. doi: 10.1007/s00436-015-4513-0.

27. Kybicová K, Schánilec P, Hulínská D, Uherková L, Kurzová Z, Spejchalová S. Detection of *Anaplasma phagocytophilum* and *Borrelia burgdorferi* sensu lato in dogs in the Czech Republic. *Vector Borne Zoonotic Dis* (2009) 9:655-61. doi: 10.1089/vbz.2008.0127.

28. Beck A, Huber D, Antolić M, Anzulović Ž, Reil I, Polkinghorne A, et al. Retrospective study of canine infectious haemolytic anaemia cases reveals the importance of molecular investigation in accurate postmortal diagnostic protocols. *Comp Immunol Microbiol Infect Dis* (2019) 65:81-7. doi: 10.1016/j.cimid.2019.05.006.

29. Pérez Vera C, Kapiainen S, Junnikkala S, Aaltonen K, Spillmann T, Vapalahti O. Survey of selected tick-borne diseases in dogs in Finland. *Parasit Vectors* (2014) 7:285. doi: 10.1186/1756-3305-7-285.

30. Pantchev N, Schaper R, Limousin S, Norden N, Weise M, Lorentzen L. Occurrence of *Dirofilaria immitis* and tick-borne infections caused by *Anaplasma phagocytophilum, Borrelia burgdorferi* sensu lato and *Ehrlichia canis* in domestic dogs in France: Results of a countrywide serologic survey. *Parasitol Res* (2009) 105:101-14. doi: 10.1007/s00436-009-1501-2.

31. Jensen J, Simon D, Escobar HM, Soller JT, Bullerdiek J, Beelitz P, et al. *Anaplasma phagocytophilum* in dogs in Germany. *Zoonoses Public Health* (2007) 54:94-101. doi: 10.1111/j.1863-2378.2007.01028.x.

32. Kohn B, Silaghi C, Galke D, Arndt G, Pfister K. Infections with *Anaplasma phagocytophilum* in dogs in Germany. *Research in Veterinary Science* (2011) 91:71-6. doi: 10.1016/j.rvsc.2010.08.008.

33. Barutzki D, De Nicola A, Zeziola M, Reule M. Seroprevalence of *Anaplasma phagocytophilum* infection in dogs in Germany. *Berl Munch Tierarztl Wochenschr* (2006) 119:342-7. PubMed ID.

34. Krupka I, Pantchev N, Lorentzen L, Weise M, Straubinger RK. Durch Zecken übertragbare bakterielle Infektionen bei Hunden: Seroprävalenzen von *Anaplasma phagocytophilum*, *Borrelia burgdorferi* sensu lato und *Ehrlichia canis* in Deutschland. *Praktischer Tierarzt* (2007) 88:776-88.

35. Kohn B, Galke D, Beelitz P, Pfister K. Clinical features of canine granulocytic anaplasmosis in 18 naturally infected dogs. *J Vet Intern Med* (2008) 22:1289-95. doi: 10.1111/j.1939-1676.2008.0180.x.

36. Chirek A, Silaghi C, Pfister K, Kohn B. Vorkommen von *Anaplasma phagocytophilum* bei Blutspenderhunden in Berlin/Brandenburg (2006–2012): retrospektive Auswertung klinischer Daten und Bedeutung für die Transfusionsmedizin. *Berl Munch Tierarztl Wochenschr* (2018):124-30. doi: 10.2376/0005-9366-17005.

37. Chirek A, Silaghi C, Pfister K, Kohn B. Granulocytic anaplasmosis in 63 dogs: clinical signs, laboratory results, therapy and course of disease. *J Small Anim Pract* (2018) 59:112-20. doi: 10.1111/jsap.12787.

38. Liesner JM, Krücken J, Schaper R, Pachnicke S, Kohn B, Müller E, et al. Vector-borne pathogens in dogs and red foxes from the federal state of Brandenburg, Germany. *Vet Parasitol* (2016) 224:44-51. doi: 10.1016/j.vetpar.2016.05.012.

39. Barth C, Straubinger RK, Sauter-Louis C, Hartmann K. Prevalence of antibodies against *Borrelia burgdorferi* sensu lato and *Anaplasma phagocytophilum* and their clinical relevance in dogs in Munich, Germany. *Berl Munch Tierarztl Wochenschr* (2012) 125:337-44. PubMed ID: 22919928.

40. Preyß-Jägeler C, Müller E, Straubinger RK, Hartmann K. Prävalenz von Antikörpern gegen *Borrelia burgdorferi*, *Anaplasma phagocytophilum* und bestimmte *Leptospira-interrogans*-Serovare bei Berner Sennenhunden. *Tierarztl Prax Ausg K Kleintiere Heimtiere* (2016) 44:77-85. doi: 10.15654/TPK-140962.

41. Schaarschmidt-Kiener D, Müller W. Diagnostic and clinical aspects of canine anaplasmosis and ehrlichiosis. *Tierarztl Prax Ausg K Kleintiere Heimtiere* (2007) 35:129-36. doi: 10.1055/s-0038-1622611.

42. Farkas R, Gyurkovszky M, Lukács Z, Aladics B, Solymosi N. Seroprevalence of some vector-borne infections of dogs in Hungary. *Vector Borne Zoonotic Dis* (2014) 14:256-60. doi: 10.1089/vbz.2013.1469.

43. Hornok S, Horváth G, Takács N, Farkas R, Szőke K, Kontschán J. Molecular evidence of a badger-associated *Ehrlichia* sp., a *Candidatus* Neoehrlichia lotoris-like genotype and *Anaplasma marginale* in dogs. *Ticks Tick Borne Dis* (2018) 9:1302-9. doi: 10.1016/j.ttbdis.2018.05.012.

44. Berzina I, Capligina V, Bormane A, Pavulina A, Baumanis V, Ranka R, et al. Association between *Anaplasma phagocytophilum* seroprevalence in dogs and distribution of *Ixodes ricinus* and *Ixodes persulcatus* ticks in Latvia. *Ticks Tick Borne Dis* (2013) 4:83-8. doi: 10.1016/j.ttbdis.2012.08.003.

45. Radzijevskaja J, Tamoliūnaitė D, Sabūnas V, Aleksandravičienė A, Paulauskas A. Prevalence and co-infection of mosquito-and tick-borne pathogens in domestic dogs suspected for canine babesiosis in Lithuania. *Biologija* (2020) 66:94-102. doi: 10.6001/biologija.v66i2.4256

46. Rymaszewska A, Małgorzata A. Molecular evidence of vector-borne pathogens coinfecting dogs from Poland. *Acta Vet Hung* (2011) 59:215. doi: 10.1556/avet.2011.008.

47. Zygner W, Górski P, Wędrychowicz H. Detection of the DNA of *Borrelia afzelii*, *Anaplasma phagocytophilum* and *Babesia canis* in blood samples from dogs in Warsaw. *Vet Rec* (2009) 164:465. doi: 10.1136/vr.164.15.465.

48. Welc-Falęciak R, Rodo A, Siński E, Bajer A. *Babesia cani*s and other tick-borne infections in dogs in central Poland. *Vet Parasitol* (2009) 166:191-8. doi: 10.1016/j.vetpar.2009.09.038.

49. Bajer A, Mierzejewska EJ, Rodo A, Bednarska M, Kowalec M, Welc-Falęciak R. The risk of vector-borne infections in sled dogs associated with existing and new endemic areas in Poland: Part 1: A population study on sled dogs during the racing season. *Vet Parasitol* (2014) 202:276-86. doi: 10.1016/j.vetpar.2013.12.033.

50. Krämer F, Schaper R, Schunack B, Połozowski A, Piekarska J, Szwedko A, et al. Serological detection of *Anaplasma phagocytophilum, Borrelia burgdorferi* sensu lato and *Ehrlichia canis* antibodies and *Dirofilaria immitis* antigen in a countrywide survey in dogs in Poland. *Parasitol Res* (2014) 113:3229-39. doi: 10.1007/s00436-014-3985-7.

51. Dzięgiel B, Adaszek Ł, Carbonero A, Łyp P, Winiarczyk M, Dębiak P, et al. Detection of canine vector-borne diseases in eastern Poland by ELISA and PCR. *Parasitol Res* (2016) 115:1039-44. doi: 10.1007/s00436-015-4832-1.

52. Víchová B, Miterpáková M, Iglódyová A. Molecular detection of co-infections with *Anaplasma phagocytophilum* and/or *Babesia canis canis* in *Dirofilaria*-positive dogs from Slovakia. *Vet Parasitol* (2014) 203:167-72. doi: 10.1016/j.vetpar.2014.01.022.

53. Čabanová V, Pantchev N, Hurníková Z, Miterpáková M. Recent study on canine vector-borne zoonoses in southern Slovakia - serologic survey. *Acta Parasitol* (2015) 60:749-58. doi: 10.1515/ap-2015-0107.

54. Egenvall AE, Hedhammar ÅA, Bjöersdorff AI. Clinical features and serology of 14 dogs affected by granulocytic ehrlichiosis in Sweden. *Vet Rec* (1997) 140:222. doi: 10.1136/vr.140.9.222.

55. Egenvall A, Bonnett BN, Gunnarsson A, Hedhammar A, Shoukri M, Bornstein S, et al. Sero-prevalence of granulocytic *Ehrlichia* spp. and *Borrelia burgdorferi* sensu lato in Swedish dogs 1991-94. *Scand J Infect Dis* (2000) 32:19-25. doi: 10.1080/00365540050164164.

56. Jäderlund KH, Egenvall A, Bergström K, Hedhammar Å. Seroprevalence of *Borrelia burgdorferi* sensu lato and *Anaplasma phagocytophilum* in dogs with neurological signs. *Vet Rec* (2007) 160:825. doi: 10.1136/vr.160.24.825.

57. Jäderlund KH, Bergström K, Egenvall A, Hedhammar Å. Cerebrospinal fluid PCR and antibody concentrations against *Anaplasma phagocytophilum* and *Borrelia burgdorferi* sensu lato in dogs with neurological signs. *J Vet Intern Med* (2009) 23:669-72. doi: 10.1111/j.1939-1676.2009.0313.x.

58. Pusterla N, Pusterla JB, Deplazes P, Wolfensberger C, Müller W, Hörauf A, et al. Seroprevalence of *Ehrlichia canis* and of canine granulocytic *Ehrlichia* infection in dogs in Switzerland. *J Clin Microbiol* (1998) 36:3460-2. doi: 10.1128/JCM.36.12.3460-3462.1998.

59. Hofmann-Lehmann R, Wagmann N, Meli ML, Riond B, Novacco M, Joekel D, et al. Detection of *Candidatus* Neoehrlichia mikurensis and other Anaplasmataceae and Rickettsiaceae in Canidae in Switzerland and Mediterranean countries. *Schweiz Arch Tierheilkd* (2016) 158:691-700. doi: 10.17236/sat00087.

60. Shaw SE, Binns SH, Birtles RJ, Day MJ, Smithson RC, Kenny MJ. Molecular evidence of tick-transmitted infections in dogs and cats in the United Kingdom. *Vet Rec* (2005) 157:645. doi: 10.1136/vr.157.21.645.

61. Juvet F, Lappin MR, Brennan S, Mooney CT. Prevalence of selected infectious agents in cats in Ireland. *J Feline Med Surg* (2010) 12:476-82. doi: 10.1016/j.jfms.2010.02.003.

62. Bergmann M, Englert T, Stuetzer B, Hawley JR, Lappin MR, Hartmann K. Prevalence of selected rickettsial infections in cats in southern Germany. *Comp Immunol Microbiol Infect Dis* (2015) 42:33-6. doi: 10.1016/j.cimid.2015.08.003.

63. Hamel D, Bondarenko A, Silaghi C, Nolte I, Pfister K. Seroprevalence and bacteremia [corrected] of *Anaplasma phagocytophilum* in cats from Bavaria and Lower Saxony (Germany). *Berl Munch Tierarztl Wochenschr* (2012) 125:163-7. doi: 10.2371/0005-9311-125-113.

64. Krücken J, Schreiber C, Maaz D, Kohn M, Demeler J, Beck S, et al. A novel high-resolution melt PCR assay discriminates *Anaplasma phagocytophilum* and *Candidatus* Neoehrlichia mikurensis. *J Clin Microbiol* (2013) 51:1958-61. doi: 10.1128/jcm.00284-13.

65. Morgenthal D, Hamel D, Arndt G, Silaghi C, Pfister K, Kempf VAJ, et al. Prävalenz von hämotrophen *Mycoplasma* spp., *Bartonella* spp. und *Anaplasma phagocytophilum* bei Katzen im Raum Berlin/Brandenburg. *Berl Munch Tierarztl Wochenschr* (2012) 125:418-27. doi: 10.2376/0005-9366-125-﻿11.
