# Supplementary figures and images for: Zoonotic Tick-Borne Pathogens in Temperate and Cold Regions of Europe—A Review on the Prevalence in Domestic Animals
Source: Front Vet Sci. 2020 Dec 10;7:604910. doi: 10.3389/fvets.2020.604910 (PMC7758354; doi:10.3389/fvets.2020.604910)

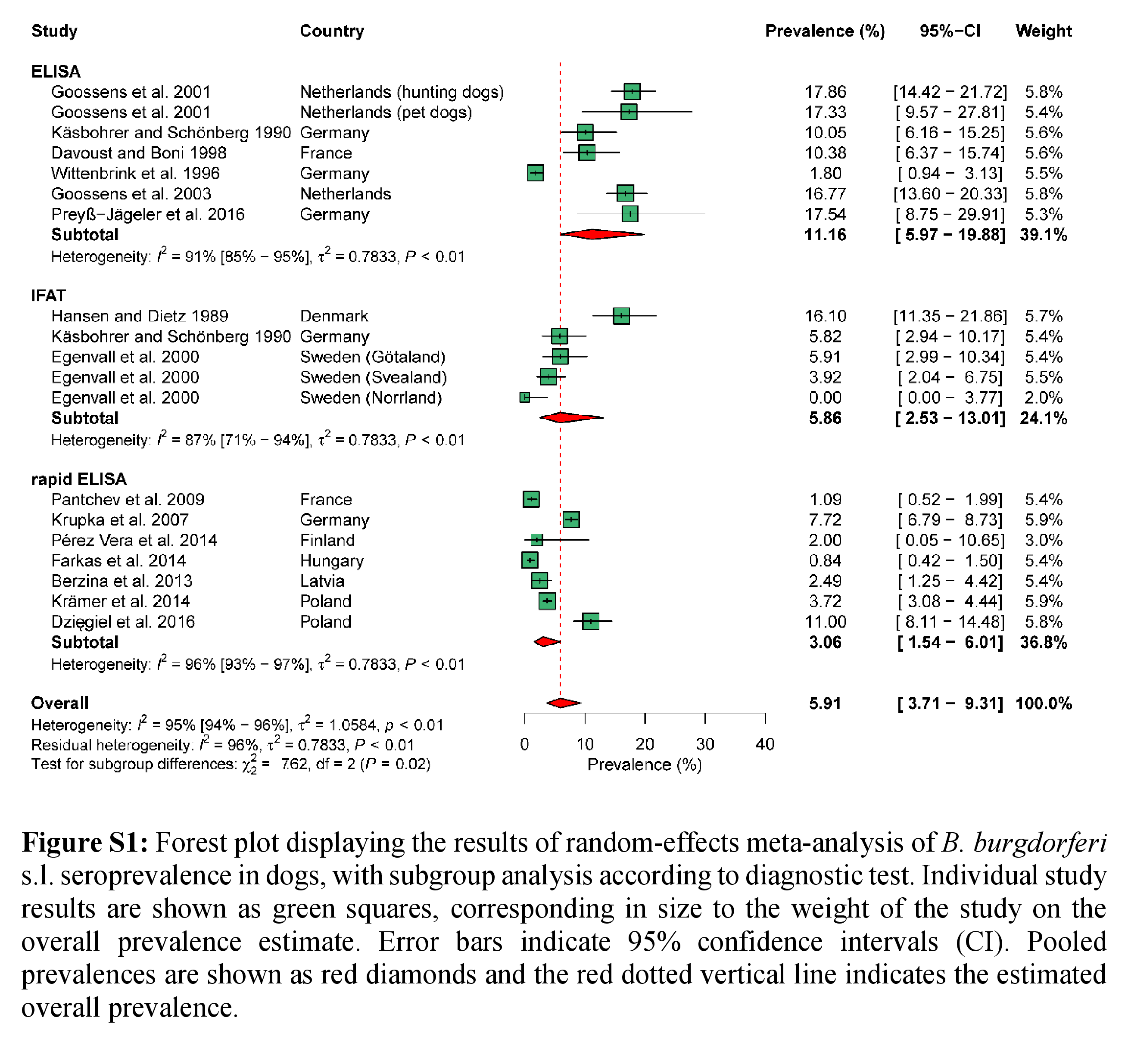

Supplement: Supplementary file 4 [file Image_1.TIF]

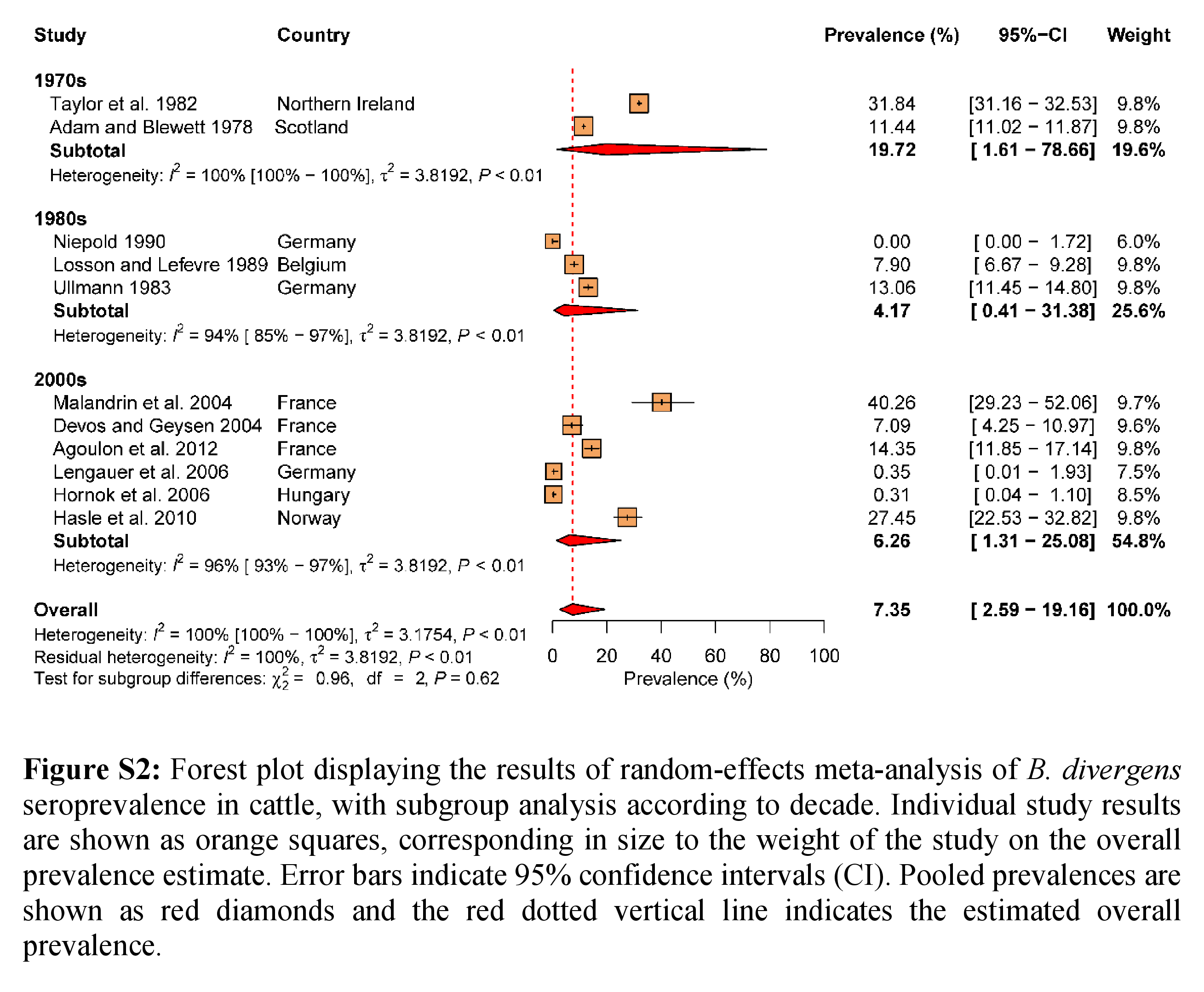

Supplement: Supplementary file 5 [file Image_2.TIF]
